# Modeling contextual effects using individual-level data and without aggregation: an illustration of multilevel factor analysis (MLFA) with collective efficacy

**DOI:** 10.1186/s12963-015-0045-1

**Published:** 2015-05-10

**Authors:** Erin C Dunn, Katherine E Masyn, William R Johnston, SV Subramanian

**Affiliations:** Psychiatric and Neurodevelopmental Genetics Unit, Center for Human Genetic Research, Massachusetts General Hospital, 185 Cambridge Street, Simches, Room 6.252, Boston, MA 02114 USA; Division of Epidemiology and Biostatistics, School of Public Health, Georgia State University, Atlanta, GA 30302 USA; Harvard Graduate School of Education, 6 Appian Way, Cambridge, MA 02138 USA; Department of Social and Behavioral Sciences, Harvard T.H. Chan School of Public Health, 677 Huntington Avenue, Kresge Building 7th Floor, 716, Boston, Massachusetts 02115 USA

**Keywords:** Multilevel, Factor analysis, Environment, Ecological, Context, Latent variable, Collective efficacy, Neighborhood

## Abstract

Population health scientists increasingly study how contextual-level attributes affect individual health. A major challenge in this domain relates to measurement, i.e., how best to measure and create variables that capture characteristics of individuals and their embedded contexts. This paper presents an illustration of multilevel factor analysis (MLFA), an analytic method that enables researchers to model contextual effects using individual-level data without using derived variables. MLFA uses the shared variance in sets of observed items among individuals within the same context to estimate a measurement model for latent constructs; it does this by decomposing the total sample variance-covariance matrix into *within*-group (e.g., individual-level) and *between*-group (e.g., contextual-level) matrices and simultaneously modeling *distinct* latent factor structures at each level. We illustrate the MLFA method using items capturing collective efficacy, which were self-reported by 2,599 adults in 65 census tracts from the Los Angeles Family and Neighborhood Survey (LAFANS). MLFA identified two latent factors at the individual level and one factor at the neighborhood level. Indicators of collective efficacy performed differently at each level. The ability of MLFA to identify different latent factor structures at each level underscores the utility of this analytic tool to model and identify attributes of contexts relevant to health.

**Electronic supplementary material:**

The online version of this article (doi:10.1186/s12963-015-0045-1) contains supplementary material, which is available to authorized users.

Population health scientists are increasingly interested in studying multilevel phenomena, or how features of the social and physical contexts in which individuals live, learn, work, and play (e.g., neighborhoods, schools, or workplaces) are associated with individual health, disease, and behavior [1,2]. A major challenge faced by multilevel researchers relates to measurement and how best to measure features of contexts and create variables that capture both the characteristics of individuals and the contexts in which they are embedded. Identifying novel measures to capture the features of contexts that may be relevant to health is an area where multilevel researchers have urged for more progress [3-8].

One of the best examples of the challenges related to and limitations of existing approaches with regards to measurement of multilevel phenomena is evident in research on collective efficacy. Collective efficacy was first articulated in a paper by Sampson and colleagues as a feature of neighborhoods that consists of two dimensions: social cohesion among neighbors (social cohesion) and neighbors’ willingness to intervene on behalf of the common good (informal social control) [9]. Since its introduction, collective efficacy has been one of the most heavily studied constructs in epidemiological and population-based research, particularly neighborhood studies, with more than 5,000 articles citing the paper introducing the concept. Collective efficacy has been found in numerous empirical studies to be positively associated with many health and developmental outcomes [9-14].

As shown in Table 1, several approaches have been used to create variables that capture collective efficacy or related contextual-level social phenomena, such as income inequality or social capital. The most popular approach has been to create a derived variable, which entails summarizing the characteristics of individuals within a group, using means, medians, proportions, or measures of dispersion (e.g., variances) or other aggregation approaches [15]. Means have been the most popular type of derived variable used in research on collective efficacy as well as other areas of multilevel research. To construct these group or contextual-level means, the major strategy has been to first average individual responses to items on a given scale; these means are then subsequently averaged across individuals living in the same context (e.g., neighborhood) to arrive at a contextual-level measure [10,14,16-19].

**Table 1:** **Approaches used to construct variables to model the effects of collective efficacy or related social-environmental variables, such as income inequality or social capital**

**Variable approach**	**Description**	**Examples**
*Derived variable*	Derived variables are created by summarizing the characteristics of individuals within a group, using means, medians, proportions, or measures of dispersion (e.g., variances) or other aggregation approaches	
Based on group-level mean	Use average individual responses to items on a given scale; these means are then subsequently averaged across individuals living in the same context (e.g., neighborhood) to arrive at a contextual-level measure.	[10,14,16,17]
Based on group-level variance	Use average individual responses to items on a given scale; the variance (or standard deviation) in these means are then examined among individuals living in the same context (e.g., neighborhood) to arrive at a contextual-level measure.	[19]
*Factor Analysis*	Capture the shared variance among an observed set of variables in terms of a potentially smaller number of unobserved constructs or latent factors.	
Single-level factor analysis	Latent factors are estimated at only one level (i.e., the individual or contextual level).	[18]
Multilevel factor analysis (MLFA)	Latent factors are estimated at two-levels of analysis. Latent factors structures can differ at each level of analysis.	[24-28]
*Hierarchical Latent Variable Model*	A special case of the 2-level MLFA that imposes stricter parameter constraints than the most general MLFA wherein latent factors are estimated at only the individual level with the factor variances decomposed into within- and between-group components.	[9,51]

A second approach has been to use factor analytic or latent variable models to determine whether multiple items should be grouped together in a common construct. Although factor analytic methods can be conducted at one or more levels of analysis (e.g., individual level, contextual level, or both), the majority of studies have focused on single-level factor analytic approaches [18]. Few studies have used latent variable approaches to study collective efficacy, even though the authors introducing the concept used a hierarchical linear latent variable modeling approach to study collective efficacy and estimate its relationship to violent crime [9].

While both derived variables and single-level factor analytic approaches are widely used and easy to construct, their use in multilevel research may be problematic in some cases. For example, there may be instances when more than one variable best represents the contextual-level phenomenon. Moreover, there may also be instances when it is misleading to assume the function of the items and how they relate to each other is the same at all levels of analysis. New approaches are therefore needed that allow researchers to model contextual effects using individual-level data when existing measurement strategies (e.g., derived variables, single-level factor analyses) are not ideal.

In an effort to expand the population health scientist’s toolkit, this paper provides an applied example of one analytic technique – multilevel factor analysis (MLFA) – that is a good alternative to existing approaches to create group or contextual-level measures. MLFA is not a new method, as it was first articulated more than 25 years ago [20-23]. However, the method has not yet been widely used, especially in population health and epidemiology. MLFA allows researchers to both model contextual effects using individual-level data without using derived variables and create variables that capture individual as well as group-level variability using one or more measures at each level of analysis (see for example [24-28]).

MLFA is part of a family of factor analytic models that seek to capture the shared variance among an observed set of variables in terms of a potentially smaller number of unobserved constructs or latent factors. Conceptually and analytically, MLFA is distinct from the other measurement approaches, including derived variables, single-level factor analyses, and hierarchical latent variable models (HLVM), which all assume the constructs of interest are the *same* at each level of analysis. Single-level exploratory (EFA) or confirmatory factor analysis (CFA) estimates latent factors at only one level (i.e., the individual or contextual level). HLVM also estimates latent factors at only one level but captures both within- and between-level variability in those factors. In contrast, MLFA allows for different latent factor structures at each level of analysis. This occurs because the MLFA decomposes the total sample variance-covariance matrix into *within*-group (i.e., individual-level, within a context) and *between*-group (i.e., contextual-level) matrices and simultaneously models *distinct* latent factor structures at each of these levels [22,29,30]. As we detail below, HLVM is a special case of MLFA. Thus, MLFA can be viewed as an analytic approach that allows the user to relax some of the potentially untenable assumptions and constraints imposed by the HLVM specification.

In this methodological demonstration, we apply MLFA to examine the underlying factor structure of items measuring collective efficacy and compare the results to the closest analytic alternative, the HLVM. Although our focus is on collective efficacy for demonstration purposes, the MLFA technique can be applied to numerous other possible contextual-level social constructs. The MLFA technique could also be extended to evaluate the measurement quality (e.g., reliability and validity) of contextual or ecological measures, including those that are directly assessed (rather than ascertained through data collected on individuals), as has been advocated by researchers concerned with “ecometrics” [6,31].

A web-based Technical Guide (see Additional file 1) is provided to guide users in implementing MLFA in MPlus. This Technical Guide is intended to guide readers on the procedures to fit and interpret results from two multilevel factor analytic models: (1) a multilevel exploratory factor analysis (ML-EFA), and (2) multilevel confirmatory factor analysis (ML-CFA).

## Methods

### Sample and study design

Data came from the Los Angeles Family and Neighborhood Survey (L.A. FANS), a longitudinal study examining the impact of neighborhoods on children’s development and well-being [32]. The study followed a stratified random sample of 3,090 households from 65 census tracts in Los Angeles County. Within each household that contained both adults and school-aged children, a randomly selected adult (RSA) was chosen, who completed surveys at Wave I (Spring 2000-Fall 2001). For the current study, we used data on perceptions of the neighborhood collected from the RSA. Our analytic sample consisted of 2,594 RSA respondents living in 65 census tracts. Respondents were primarily female (69.1%), Latino(a) (59.5%), and non-home owners (59.4%), with a mean age of 38.8 years (sd = 13.6).

### Measures

#### Collective efficacy

Based on previous work [9], collective efficacy was measured using 10 items that captured both perceived neighborhood informal social control and social cohesion [10].

Social cohesion was measured using seven items (refer to items 1–7 in Table 2) rated on a five-point scale (1 = strongly agree to 5 = strongly disagree). Informal social control was measured using three items (refer to items 8–10 in Table 2) rated on a five-point scale (1 = very unlikely to 5 = very likely) indicating how likely the respondent would be to intervene if they witnessed these three events.

**Table 2:** **Intraclass Correlation Coefficients (ICC) for indicator variables in the Los Angeles Family and Neighborhood Study (LAFANS) n = 2594**

	**Intraclass correlation coefficient**		
	**Total sample**	**Sample one**	**Sample two**
**Indicator variables**	***N = 2594***	***n = 1291***	***n = 1303***
1…this is a close-knit neighborhood	0.083	0.112	0.121
2…there are adults that kids look up to	0.198	0.253	0.216
3…people around here are willing to help their neighbors	0.133	0.142	0.174
4…people in this neighborhood generally don’t get along with each other	0.149	0.148	0.178
5…adults watch out that kids are safe	0.085	0.112	0.089
6…people in this neighborhood do not share the same values	0.120	0.174	0.114
7…people in this neighborhood can be trusted	0.203	0.198	0.254
8…children were skipping school and hanging out on a street corner	0.104	0.131	0.125
9…children were spray-painting graffiti on a local building	0.262	0.299	0.273
10…children were showing disrespect to an adult	0.062	0.093	0.090

ICC refers to the proportion of variance in the indicator variable that is due to differences across neighborhoods. Neighborhoods were defined here as census tracts.

Items number 4 and 6 were reverse coded.

### Statistical analysis

We used multilevel factor analysis (MLFA), a method that models the responses for person *i* in cluster *j* (e.g., neighborhood) to a set of *M* items (or indicator variables), denoted **y**_*ij*_ = (*y*_1*ij*_, …, *y*_*Mij*_), as a function of both individual-level (i.e., *w*ithin-group or “Level 1”) and neighborhood-level (i.e., *b*etween-group or “Level 2”) factors, represented by **η**_*W*_ and **η**_*B*_, respectively.

The within-group model is given by

1 $$ {\mathbf{y}}_{ij}={\boldsymbol{\upnu}}_j+{\boldsymbol{\Lambda}}_W{\boldsymbol{\upeta}}_{Wij}+{\boldsymbol{\upvarepsilon}}_{ij}, $$

where **ν**_*j*_ is a vector of the neighborhood *j*’s mean responses for each of the *M* items for the population of individuals embedded in neighborhood *j*; **η**_*Wij*_ is a vector of individual *i*’s values for the individual-level factors, with *Ε*(**η**_*W*_) = **0** and Var(**η**_*W*_) = **ψ**_*W*_ ; **Λ**_*W*_ is a matrix of factor loadings describing the relationships between the individual-level factors, **η**_*W*_, and the indicator variables, **y**_*ij*_; and **ε**_*ij*_ is the residual for individual *i* in neighborhood *j*, with *Ε*(**ε**) = **0** and Var(**ε**) = **θ**. Typically, with continuous *y*s, the residuals and factors are specified to be normally distributed, with all residuals uncorrelated with each other and with the factors.

The between-group model is given by

2 $$ {\boldsymbol{\upnu}}_j=\boldsymbol{\upgamma} +{\boldsymbol{\Lambda}}_B{\boldsymbol{\upeta}}_{Bj}+{\boldsymbol{\upzeta}}_j, $$

where **γ** is a vector of overall means for the *M* items; **η**_*Bj*_ is a vector of neighborhood *j*’s values for the group-level factors, with *Ε*(**η**_*B*_) = **0** and Var(**η**_*B*_) = **ψ**_*B*_; **Λ**_*B*_ is a matrix of factor loadings describing the relationships between the group-level factors, **η**_*B*_, and the group-level random intercept indicators, **ν**_*j*_; and **ζ**_*j*_ is the residual for neighborhood *j*, with *Ε*(**ζ**) = **0** and Var(**ζ**) = **σ**. Like the within-group model, the residuals and factors are specified to be normally distributed, with all residuals uncorrelated with each other and with the factors.

**Figure 1:**
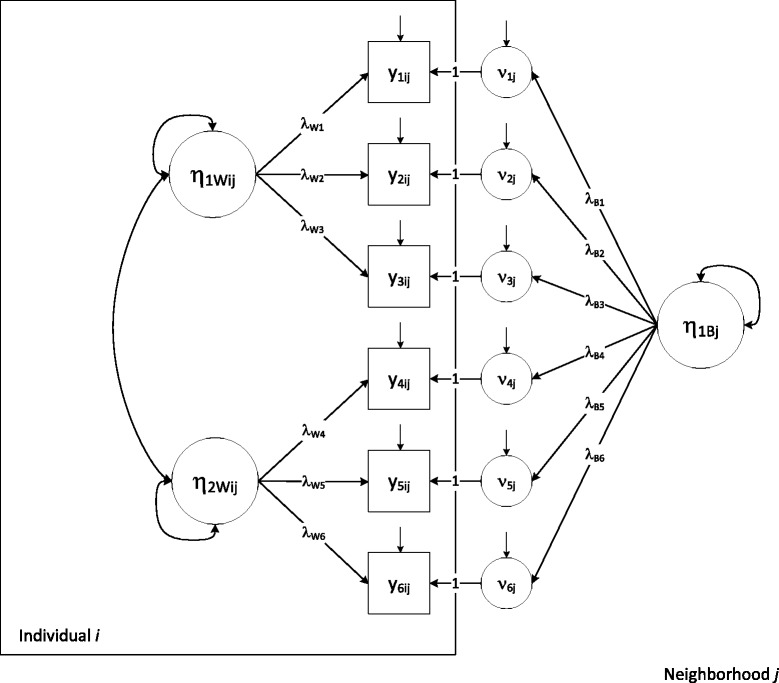
Path diagram for a hypothetical 6-item multilevel confirmatory factor analysis (ML-CFA) with two individual-level and one neighborhood-level factors.

Substituting Equation 2 into Equation 1 yields a single combined model:

3 $$ {\mathbf{y}}_{ij}=\boldsymbol{\upgamma} +{\boldsymbol{\Lambda}}_W{\boldsymbol{\upeta}}_{Wij}+{\boldsymbol{\Lambda}}_B{\boldsymbol{\upeta}}_{Bj}+{\boldsymbol{\upzeta}}_j+{\boldsymbol{\upvarepsilon}}_{ij}, $$

showing that the observed responses at the individual level are specified as distinct effects of both individual- and group-level factors. These effects are depicted in Figure 1 by a path diagram for a hypothetical six-item MLFA with two within-group and one between-group factors. The variables (observed in squares and latent in circles) within the “Individual *i*” box are variables that vary across each individual embedded in neighborhood *j*. The variables outside the “Individual *i*” box and within the “Neighborhood *j*” box vary across each neighborhood, but are constant for all individuals within a given neighborhood. The individual-level and neighborhood-level residuals are represented by the small arrows pointing to the observed *ys* and the neighborhood-level random intercept, respectively.

The model described in Equations 1 and 2 can be extended to non-continuous (e.g., binary, ordinal, count, etc.) indicator variables using a generalized linear model formulation. Briefly (and as outlined in greater detail in [33,34]), any vector of indicator variables, **y**_*ij*_, can be expressed as the sum of the individual expected values, **μ**_*ij*_ and the individual residuals, **ε**_*ij*_; that is,

4 $$ {\mathbf{y}}_{ij}={\boldsymbol{\upmu}}_{y_{ij}}+{\boldsymbol{\upvarepsilon}}_{ij}. $$

The distribution of the residuals is chosen to correspond to the measurement scale of the observed indicators, e.g., a Bernoulli distribution for binary indicators. A link function, *g*, then relates the individual expected values to a linear combination of the latent factors; that is,

5 $$ g\left({\boldsymbol{\upmu}}_{y_{ij}}\right)={\boldsymbol{\upnu}}_j+{\boldsymbol{\Lambda}}_W{\boldsymbol{\upeta}}_{Wij}. $$

The between-group model remains the same. In the case of continuous approximately normally distributed observed outcomes, the usual specification is the identity link function, resulting in straightforward linear regressions relating the observed variables to the latent factor. In the case of binary indicators, one might choose a logit link function, resulting in logistic regressions relating the observed categorical indicators to the latent factors. In the case of an observed ordinal response scale, as with our indicators of collective efficacy, we used the ordinal probit link function [35]. All models were estimated via weighted least squares using a diagonal weight matrix with standard errors and mean- and variance-adjusted chi-square test statistics that used a full weight matrix (WLSMV).

To showcase the MLFA approach, we conducted our analyses in four steps. First, we calculated intraclass correlation coefficients (ICCs) for each item. These ICCs provide information about the proportion of variance in each item that is due to differences between neighborhoods. Second, we used polychoric correlations (where each correlation is a measure of the pairwise association for two ordinal variables, which rests upon the assumption of an underlying joint continuous distribution) to examine the strength, direction, and magnitude of the associations among the items. We examined these associations in two correlation matrices: (1) the within-level (individual) matrix; and (2) the between-level (neighborhood) matrix. Third, we randomly split the sample into two equally sized subsamples and conducted a multilevel exploratory analysis (ML-EFA) with one subsample and a confirmatory analysis (ML-CFA) with the other. An EFA is ideal to use in situations when researchers lack hypotheses concerning the number of latent factors underlying an item set or what the relationships are between each factor and the items; a CFA is more appropriate when researchers have hypotheses regarding the number of factors and the factor-item relationships or are seeking to test the validity of a theoretical model [36,37]. Both techniques are shown here for illustration purposes.

Finally, we fit the hierarchical latent variable model (HLVM) outlined by Sampson et al. [9] as a comparison. The HLVM is a special case of the MLFA, where the factor measurement model is the same (i.e., same number of factors, same loading patterns, and same loading values) at the within- and between-group models and there is no between-group item-specific residual. HLMV can also be seen as an extension of a single-level factor analysis, where the overall factor variance-covariance structure is comprised of within- and between-group variance-covariance components. The important distinction between the MLFA and HLVM is that the factors in the HLVM are only defined at the within-level while in the MLFA there are *distinct* factors defined at *both* the within- and between-level models. For the HLVM, the within-group is the same as for the MLFA, as given in Equation (1). The between-group model is given by

6 $$ {\boldsymbol{\upnu}}_j=\boldsymbol{\upgamma} +{\boldsymbol{\Lambda}}_W{\boldsymbol{\upeta}}_{Bj}. $$

Substituting Equation (6) into Equation (1) yields a single combined model for the HLVM:

7 $$ {\mathbf{y}}_{ij}=\boldsymbol{\upgamma} +{\boldsymbol{\Lambda}}_W\left({\boldsymbol{\upeta}}_{Wij}+{\boldsymbol{\upeta}}_{Bj}\right)+{\boldsymbol{\upvarepsilon}}_{ij}, $$

where **γ** is a vector of overall means for the *M* items; **η**_*Wij*_ and **η**_*Bj*_ capture within-group across-person variability and between-group variability, respectively, in a set of latent factors, **η**, with *Ε*(**η**) = **0** and Var(**η**) = **ψ**_*W*_ + **ψ**_*B*_ ; **Λ**_*W*_ is a matrix of factor loadings describing the relationships between the factors, **η**, and the indicator variables, **y**_*ij*_; and **ε**_*ij*_ is the residual for individual *i* in neighborhood *j*, with *Ε*(**ε**) = **0** and Var(**ε**) = **θ**. The HLVM can be more simply written as

8 $$ \begin{array}{l}{\mathbf{y}}_{ij}=\boldsymbol{\upgamma} +\boldsymbol{\Lambda} {\boldsymbol{\upeta}}_{ij}+{\boldsymbol{\upvarepsilon}}_{ij},\\ {}{\boldsymbol{\upeta}}_{ij}={\boldsymbol{\upalpha}}_j+{\boldsymbol{\upxi}}_{ij},\end{array} $$

showing that the observed indicators are a function of only individual-level factors with the variance-covariance of those factors explicitly decomposed by the model into within-group and between-group variance components. As with the MLFA, the HLVM can use a generalized linear model approach to specify the relationships between the items and the factor in the case of non-continuous item responses. The specific HLVM model used by Sampson et al. [9], expressed as a three-level model with items nested within persons nested within clusters, imposes the additional constraints of all factor loadings being fixed at one and all item residual variances constrained to be equal.

We conducted all analyses using Mplus software version 7. Mplus handles missing data under the missing at random assumption (MAR) using the WLSMV estimator, which allows missingness to be a function of the observed covariates, but not observed outcomes, as is the case for full information maximum likelihood (FIML). When there are no covariates in the model, as is the case here, this is analogous to pairwise present analysis [38,39]. Analyses also included sampling weights to adjust for non-response and the unequal probability of selection of neighborhoods and households into the sample. Across all models, we evaluated goodness-of-fit using the model chi-square test, normed comparative fit index (CFI; [40]), root mean square error of approximation (RMSEA; [41]), and the standardized root mean square residual (SRMR; [38]). These statistics provide information about how well the model-estimated population correlations reproduce the sample correlations. Acceptable model fit was determined by a non-significant chi-square test, CFI values greater than 0.95, and RMSEA and SRMR values below 0.10 [42]. The CFI, RMSEA, and SRMR values were given more emphasis than the chi-square test, as the chi-square test statistic is often significant (implying there is significant misfit of the model to the data) when the sample size is large. In the MLFA, an SRMR is provided at both the within and between level. As there are no established guidelines for interpreting the SRMR at the between level, we considered the guidelines that are typically applied for single-level analyses (≤0.10). We also examined the residuals for the between-level correlation matrix, which are an indicator of model fit.

Of note, there are alternative statistical software packages, such as MLwiN or MLwiN via Stata, that can be used to estimate MLFA models. Readers interested in fitting the MLFA using MLwiN are referred to the MLwiN website: http://www.bristol.ac.uk/cmm/software/mlwin/. In addition, the MLFA method can also be fit using Markov chain Monte Carlo (MCMC) methods. Such Bayesian estimation procedures may provide a particularly good alternative to maximum likelihood methods in instances when maximum likelihood is too computationally intensive or when there are some instances of a small number of individuals per cluster or when there are a small number of overall clusters [21].

## Results

### Intraclass correlation coefficients (ICC)

ICC estimates ranged from small to large in magnitude and were generally equivalent across our split samples (Table 2). In the total sample, the largest estimated ICC (0.262) was for the item “children were spray-painting graffiti on a local building.” The lowest ICC in the total sample (0.062) was for “children were showing disrespect to an adult.” Thus, most of the variability in these items was due to differences across individuals *within* rather than *between* neighborhoods. However, there was considerable variability among the indicators as to the proportion of variation explained between neighborhoods. This suggests that neighborhood-level variation is not uniform across indicators and that for some indicators, neighborhood-level influences may be more important.

### Correlations

As shown in Tables 3 and 4, the within level (individual) and between level (neighborhood) had different correlation structures. While the average absolute correlation value at the within level was 0.304 (range r = 0.093 to r = 0.557), the average absolute correlation value at the between level was higher (average = 0.685; range r = 0.205 to r = 0.934). Some items also had markedly differently correlations at each level. For example, the items “people here do not get along with each other” and “people would intervene if children were spray painting graffiti” had a very strong correlation at the between-level (r = 0.858), but a weak correlation at the within-level (r = 0.239). These finding suggest the item-to-item relationships differ across the two levels of analysis (within- and between-level).

**Table 3:** **Correlations among indicators at the within-level**

		**1**	**2**	**3**	**4**	**5**	**6**	**7**	**8**	**9**	**10**
1	CLOSEKNIT	1.000									
2	ADULTS	0.461	1.000								
3	HELP	0.483	0.467	1.000							
4	ALONG	0.210	0.310	0.368	1.000						
5	SAFE	0.395	0.377	0.458	0.240	1.000					
6	VALUES	0.153	0.093	0.165	0.321	0.141	1.000				
7	TRUST	0.408	0.422	0.528	0.309	0.487	0.234	1.000			
8	SKIP	0.256	0.207	0.296	0.174	0.333	0.124	0.358	1.000		
9	GRAFFITI	0.219	0.239	0.283	0.212	0.358	0.163	0.294	0.557	1.000	
10	DISRESPECT	0.287	0.202	0.285	0.194	0.261	0.125	0.278	0.470	0.476	1.000

CLOSEKNIT = this is a close-knit neighborhood; ADULTS = there are adults that kids look up to; HELP = people here are willing to help their neighbors; ALONG = people here don’t get along with each other; SAFE = adults watch out that kids are safe; VALUES = people here do not share the same values; TRUST = people in this neighborhood can be trusted; SKIP = people would intervene if children were skipping school and hanging out on the corner; GRAFFITI = people would intervene if children were spray-painting graffiti; DISRESPECT = people would intervene if children were showing disrespect to an adult. Items 4 and 6 were reverse coded.

These correlations were taken from the sample used for the multilevel exploratory factor analysis (ML-EFA).

**Table 4:** **Correlations among indicators at the between-level**

		**1**	**2**	**3**	**4**	**5**	**6**	**7**	**8**	**9**	**10**
1	CLOSEKNIT	1.000									
2	ADULTS	0.735	1.000								
3	HELP	0.773	0.862	1.000							
4	ALONG	0.593	0.758	0.855	1.000						
5	SAFE	0.749	0.853	0.897	0.902	1.000					
6	VALUES	0.561	0.620	0.668	0.754	0.705	1.000				
7	TRUST	0.742	0.842	0.870	0.834	0.934	0.653	1.000			
8	SKIP	0.826	0.641	0.731	0.677	0.765	0.650	0.697	1.000		
9	GRAFFITI	0.729	0.858	0.870	0.857	0.865	0.725	0.823	0.757	1.000	
10	DISRESPECT	0.489	0.205	0.478	0.316	0.254	0.257	0.320	0.480	0.382	1.000

CLOSEKNIT = this is a close-knit neighborhood; ADULTS = there are adults that kids look up to; HELP = people here are willing to help their neighbors; ALONG = people here don’t get along with each other; SAFE = adults watch out that kids are safe; VALUES = people here do not share the same values; TRUST = people in this neighborhood can be trusted; SKIP = people would intervene if children were skipping school and hanging out on the corner; GRAFFITI = people would intervene if children were spray-painting graffiti; DISRESPECT = people would intervene if children were showing disrespect to an adult. Items 4 and 6 were reverse coded.

These correlations were taken from the sample used for the multilevel exploratory factor analysis (ML-EFA).

### Multilevel factor analysis (MLFA) results

#### Multilevel exploratory factor analysis (ML-EFA)

The final ML-EFA model, which was selected based on good model-data consistency, parsimony, and interpretability, had two within-level factors and one between-level factor (Table 5). In this factor solution, the largest factor loadings for each item at the within level (0.418 to 0.773) and between level (0.462 to 0.972) ranged from moderate to high. In addition to good overall model fit, as evidenced by the CFI of 0.947 and RMSEA of 0.059, this solution also had excellent model fit specifically at the within and between levels, as shown in the SRMR values at each level 0.039 and 0.068, respectively. In contrast, the next best fitting model – the two factor within and two-factor between model – had a good overall fit (SRMR_within_ = 0.039; SRMR_between_ = 0.045). However, the second between-level factor had only one significantly loading item (refer to page 21 of the online Technical Guide.

**Table 5:** **Factor loadings of indicators for the multi-level exploratory factor analysis (ML-EFA)**

	**Within-level**		**Between-level**
	**Factor 1**	**Factor 2**	**Factor 1**
1…this is a close-knit neighborhood	**0.618**	0.030	**0.797**
2…there are adults that kids look up to	**0.642**	−0.034	**0.833**
3…people around here are willing to help their neighbors	**0.735**	0.038	**0.935**
4…people in this neighborhood generally don’t get along with each other	**0.418**	−0.008	**0.931**
5…adults watch out that kids are safe	**0.630**	0.035	**0.972**
6…people in this neighborhood do not share the same values	0.297	0.015	**0.668**
7…people in this neighborhood can be trusted	**0.773**	−0.046	**0.924**
8…children were skipping school and hanging out on a street corner	0.121	**0.662**	**0.823**
9…children were spray-painting graffiti on a local building	0.001	**0.711**	**0.917**
10…children were showing disrespect to an adult	−0.010	**0.723**	**0.462**

χ^2^ = 337.222; df = 61; p-value < 0.00001; CFI = 0.947; RMSEA = 0.059; SRMRwithin = 0.039; SRMRbetween = 0.068.

All factor loadings in an EFA are standardized.High EFA loadings appear in bold.

Items 4 and 6 were reverse coded.

Beyond its empirical fit, the ML-EFA solution was also aligned with prior theory. At the within level, the first factor mapped on to the construct social cohesion and the second factor mapped on to the construct informal social control, as described by others [9,10]. At the between level, the indicator variables only supported one overarching factor, which has previously been labeled as collective efficacy [9,10]. Interestingly, the sixth item (people in this neighborhood do not share the same values) did not load significantly on either factor at the within level, but had a significant factor loading at the between level. This finding illustrates that indicator variables can perform differently at each level of analysis and therefore items should only be removed from a MLFA if they are determined not to function at both levels of analysis.

The first and second within-level factors were moderately correlated (r = 0.521). The communalities, or item-specific R^2^ values, which refer to the proportion of an indicator’s total variance accounted for by the factor solution, ranged at the within level from a low of 8.4% (for respondents’ rating of people in the neighborhood sharing the same values) to a high of 57.1% (for respondents’ rating of people’s willingness to help neighbors) at the within level. At the between level, the communalities were higher across the items, ranging from a low of 21.4% (for neighborhoods’ collective tendency to intervene if children show disrespect to an adult) to a high of 94.4% (for neighborhoods’ collective tendency to watch out that kids are safe).

#### Multilevel confirmatory factor analysis (ML-CFA)

The ML-EFA results from the first subsample were cross-validated using ML-CFA for the second subsample. As shown in Table 6, the fit of the ML-CFA model was good (CFI = 0.903; RMSEA = 0.079; SRMR_within_ = 0.054; SRMR_between_ = 0.073). By and large, factor loadings in the ML-CFA were similar to the ML-EFA.

**Table 6:** **Standardized factor loadings of items for the Multi-Level Confirmatory Factor Analysis (ML-CFA)**

	**Within-level**		**Between-level**
	**Factor 1**	**Factor 2**	**Factor 1**
1…this is a close-knit neighborhood	0.622		0.774
2…there are adults that kids look up to	0.631		0.824
3…people around here are willing to help their neighbors	0.701		0.857
4…people in this neighborhood generally don’t get along with each other	0.474		0.828
5…adults watch out that kids are safe	0.649		0.819
6…people in this neighborhood do not share the same values	0.266		0.807
7…people in this neighborhood can be trusted	0.681		0.897
8…children were skipping school and hanging out on a street corner		0.724	0.667
9…children were spray-painting graffiti on a local building		0.769	0.928
10…children were showing disrespect to an adult		0.613	0.353

χ^2^ = 629.816; df = 69; p-value < 0.00001; RMSEA = 0.079; CFI = 0.903; SRMR_within_ = 0.054; SRMR_between_ = 0.073.

Items 4 and 6 were reverse coded.

We also ran an alternative ML-CFA specification with the constraints imposed by the Sampson et al. version of the HLVM described earlier. The overall fit of this model was markedly worse than the ML-CFA without these restrictions (χ^2^ = 1445.265; df = 86; p-value < 0.001; RMSEA = 0.110; CFI = 0.766; SRMR_within_ = 0.095; SRMR_between_ = 0.325), suggesting that a more restricted model lacked the model-data consistency observed with the less restrictive ML-CFA. Of note, a single-level factor analysis, which is the equivalent of adding to the HLVM a further constraint of zero between-level factor variance, would have a poorer fit than the HLVM. Although not the case here, it is possible that for another dataset, the HLVM specification could fit equivalent to the MLFA. Such a finding would suggest that the data do not support a different factor structure at the within and between-group levels, and the HLVM could be favored as a more parsimonious model. A researcher, however, would not be able to make this determination without comparing the HLVM to the MLFA.

## Discussion

This methodological demonstration of MLFA to collective efficacy shows that use of either simple aggregation methods, in the form of derived variables, or single-level factor analyses, may not be the best way to construct contextual-level variables from individual-level data. We arrived at this conclusion based on three sets of results. First, we found that ICC values were not the same for every item; some items showed quite high neighborhood-level variation and others showed very little. The lack of uniformity in between-neighborhood variation across these items suggests neighborhood context may have differing levels of salience across this set of items and that not all items should be treated equally in terms of their importance to understanding neighborhoods.

Second, the correlation structure of the items was different across the individual (within) and neighborhood (between) levels. Specifically, the correlation among items was much higher at the between level than the within. Moreover, how the items related to each other also differed across levels; some items had high correlations at one level and modest correlations at the other. These findings provided an initial sign that there may be different factor structures at the two levels of analysis.

Third, when we ran the MLFA, we found that the best-fitting model was one that modeled collective efficacy as a two dimensional construct at the within level, consisting of the two latent constructs informal social control and social cohesion, and a one dimensional construct at the between level, consisting of collective efficacy. This two-factor within and one-factor between model was confirmed in the ML-CFA. Imposing an identical factor structure at both levels resulted in a worse-fitting model, particularly when we imposed a set of stricter constraints described in the original paper introducing collective efficacy [9]. While the stricter constraints may be reasonable and could be supported by the data in some cases, there may be instances, such as the case here, where the items were not all equally good indicators of collective efficacy and thus imposing equal factor loadings and equal residual variances constraints was not consistent with the observed data. We also found that the items performed differently in terms of their factor loadings at the within compared to between level. For example, the item “people in this neighborhood do not share the same values” did not load at the within level, but loaded at the between. Taken together, the results of the current study suggest that collective efficacy, and perhaps other social constructs, can have very different meanings at each level of analysis and are perhaps most appropriately studied at the neighborhood level as one overarching construct and not divided into its two dimensions, informal social control and social cohesion, as has been done in some prior studies (see for example [13,43]).

Our study has the following limitations. The measure of collective efficacy was not identical to the original measure [9]. It is possible our results would have been different had we used a different measure of collective efficacy. The number of neighborhoods in this study (n = 65) was also small relative to other studies. Moreover, our definition of neighborhoods was based on an administrative definition (i.e., Census tract), which may not adequately reflect meaningful geographic boundaries that represent distinct social experiences or cultures [44,45]. Though an imperfect measure to define neighborhoods, Census tracts are most commonly used in multilevel research in the United States [8].

Finally, the MLFA technique is, of course, not without its limitations. For example, it can be computationally intensive. Most software also only allow for two-level structures. In spite of these challenges, results of our analysis underscore the potential utility of MLFA and suggest that using other more easily implemented approaches, such as single-level factor analyses, may not be ideal. As we showed, the MFLA method revealed different latent factor structures at each level of analysis. Our results also demonstrated that imposing a simpler factor structure, with identical factor structures at each level, was not consistent with the data and resulted in a poorer-fitting model.

Results of this study have several important implications for measuring social environments potentially linked to health. Multilevel researchers have lamented the lack of progress in identifying novel measurement tools to characterize contextual-level constructs and as a result have called for new approaches [3-8]. Although more work is needed, results of the current study suggest that MLFA may be a promising method to construct variables from individual-level data for use in multilevel analyses. The MLFA technique allows researchers to use individual-level items to construct measures of the social context using a more flexible approach than other types of hierarchical models. The MLFA approach can also be easily applied with survey data, which remains the most common and cost effective type of data collected. Moreover by using MLFA, researchers establish the measurement model necessary for estimating a multilevel structural equation model (ML-SEM), where direct and indirect effects between latent variables, covariates, and individual items, existing at two or more levels of analysis, are examined [42,46,47]. Although still not widely used in epidemiology or population health, SEM models are an alternative to traditional techniques that can be used for exploratory or hypothesis-generating purposes [48] or to test more complex relationships between a set of variables [49,50].

In conclusion, our results suggest MLFA is a promising alternative to using derived variables and single-level factor analytic approaches. Future studies are warranted to validate the current results in relation to collective efficacy and extend the MLFA technique to other dimensions of the neighborhood environment as well as other social contexts that influence health.

## Additional file

Additional file 1:
**Technical Appendix for the article: Modeling contextual effects using individual-level data and without aggregation: an illustration of multilevel factor analysis (MLFA) with collective efficacy.**
