# Supplementary material for: Modeling contextual effects using individual-level data and without aggregation: an illustration of multilevel factor analysis (MLFA) with collective efficacy
Source: Popul Health Metr. 2015 May 10;13:12. doi: 10.1186/s12963-015-0045-1 (PMC4445268; doi:10.1186/s12963-015-0045-1)
Supplement: Additional file 1: — Technical Appendix for the article: Modeling contextual effects using individual-level data and without aggregation: an illustration of multilevel factor analysis (MLFA) with collective efficacy. [file 12963_2015_45_MOESM1_ESM.docx]

**Additional file 1**

From the Article**:** Modeling contextual effects using individual-level data and without aggregation: An illustration of multilevel factor analysis (MLFA) with collective efficacy

**Introduction**

This appendix is intended to guide readers on the procedures to fit and interpret results from two multilevel factor analytic models: (1) a multilevel exploratory factor analysis (ML-EFA), and (2) multilevel confirmatory factor analysis (ML-CFA). Our illustration uses data analyzed in the paper by <BLIND FOR REVIEW> noted above. We used MPlus version 7 for all analyses (Muthen & Muthen, 1998-2012).

In our analysis, all data manipulation (e.g., recoding variables, etc.) was performed prior to importing the data into MPlus. For ease of implementation, we recommend all data manipulation occur in other programs (e.g., SAS, Stata, SPSS) outside of MPlus. Readers interested in specific data manipulation capabilities should refer to the MPlus manual (Muthen & Muthen, 2012).

It is important to note that in the interest of parsimony, we do not intepret every piece of the input statements or the output. Specifically, we only present the results of the most relevant models from the ML-EFA and ML-CFA, drastically reducing the length of the output. In addition, we assume readers will have some familiarity with MPlus and with factor analytic approaches. Readers interested in learning about factor analysis are referred elsewhere (Bartholomew, 2011; Kline, 2011).

**Multilevel Exploratory Factor Analysis (ML-EFA) Syntax**

Mplus VERSION 7

MUTHEN & MUTHEN

INPUT INSTRUCTIONS

Title:

EFA for Multi-level Factor Analysis

Data:

File is ML_EFA_CFA_06_27.dat ;

Variable:

Names are caseid SAMPID_N hhid pid RSA_TYPE wgtrsa

wgtadlt wgtpcg closekni adults help along

safe values trust AB6_8 AB6_9 CLOSEKNI0

skip graffiti disrespe AB8_1 AB8_2 AB9

AB11_1 AB11_2 AB11_3 AB12 AB13 AB14

tractx sample1 ALONG_r VALUES_r AGE_YR sex

RB1 RB2_1 RB2_2 RB2_3 RB2_4 RB2_5

RB2_6 AJ5 movsince HA18_1 ;

Prior to importing the data into Mplus, we created a split sample, denoted by the variable “sample1”. Specifically, a random 50% of individuals from each cluster were given a value of 1 on this variable and the other 50% a value of 0. The EFA was conducted on one subsample and the CFA with the other. Here we specify in the USEOBSERVATIONS command that only individuals with a value of “1” on sample1 are included.

Missing are . ;

USEOBSERVATIONS = sample1 == 1;

USEVARIABLES = closekni adults help along_r

safe values_r trust skip graffiti disrespe;

The USEVARIABLES only includes the 10 items involved in the EFA.

CATEGORICAL = closekni adults help along_r

All of the items are 5-point likert scales, so they need to be identified as categorical.

safe values_r trust skip graffiti disrespe;

CLUSTER = tractx;

CLUSTER refers to the cluster variable (tractx = Census tract ID).

weight = WGTADLT;

Here we are telling Mplus to conduct a multilevel EFA with between 1 and 5 factors at both the within and between levels. Mplus will attempt to model every possible combination of factor structures (e.g., one factor within, one factor between; one factor within, two factors between, etc). The “uw” and “ub” are included to ask for unstructured models with no factors at each level. Since the data are categorical, we use the WLSMV estimator.

Analysis:

Type = twolevel efa 1 5 uw 1 5 ub;

estimator=wlsmv;

PLOT:

The plot2 option gives us scree plots for both the within and between levels.

Type = plot2;

OUTPUT:

MODINDICES provides information on how the model might be improved if it were to be modified in some way.  SAMPSTAT provides sample descriptive statistics. The SVALUES option will output parameter estimates that can be used as start values in subsequent models.

modindices sampstat svalues;

**Multilevel Exploratory Factor Analysis (ML-EFA) Results**

SUMMARY OF ANALYSIS

Number of groups 1

Number of observations 1291

Number of dependent variables 10

Number of independent variables 0

Number of continuous latent variables 0

Observed dependent variables

Binary and ordered categorical (ordinal)

CLOSEKNI ADULTS HELP ALONG_R SAFE VALUES_R

TRUST SKIP GRAFFITI DISRESPE

Variables with special functions

Cluster variable TRACTX

Weight variable (cluster-size scaling)

WGTADLT

Estimator WLSMV

Rotation GEOMIN

Row standardization CORRELATION

Type of rotation OBLIQUE

Epsilon value Varies

Optimization Specifications for the Quasi-Newton Algorithm for

Continuous Outcomes

Maximum number of iterations 1000

Convergence criterion 0.100D-05

Optimization Specifications for the EM Algorithm

Maximum number of iterations 500

Convergence criteria

Loglikelihood change 0.100D-02

Relative loglikelihood change 0.100D-05

Derivative 0.100D-02

Optimization Specifications for the M step of the EM Algorithm for

Categorical Latent variables

Number of M step iterations 1

M step convergence criterion 0.100D-02

Basis for M step termination ITERATION

Optimization Specifications for the M step of the EM Algorithm for

Censored, Binary or Ordered Categorical (Ordinal), Unordered

Categorical (Nominal) and Count Outcomes

Number of M step iterations 1

M step convergence criterion 0.100D-02

Basis for M step termination ITERATION

Maximum value for logit thresholds 10

Minimum value for logit thresholds -10

Minimum expected cell size for chi-square 0.100D-01

Maximum number of iterations for H1 2000

Convergence criterion for H1 0.100D-03

Optimization Specifications for the Exploratory Factor Analysis

Rotation Algorithm

Number of random starts 30

Maximum number of iterations 10000

Derivative convergence criterion 0.100D-04

Optimization algorithm FS

Integration Specifications

Type STANDARD

Number of integration points 7

Dimensions of numerical integration 2

Adaptive quadrature ON

Link PROBIT

Cholesky ON

Input data file(s)

ML_EFA_CFA_06_27.dat

Input data format FREE

These ICCs represent the between-level variance divided by the total variance for each item. Near-zero ICCs suggest there is minimal between-neighborhood variance on the item.

SUMMARY OF DATA

Number of clusters 65

Average cluster size 19.862

Estimated Intraclass Correlations for the Y Variables

Intraclass Intraclass Intraclass

Variable Correlation Variable Correlation Variable Correlation

CLOSEKNI 0.112 ADULTS 0.253 HELP 0.142

ALONG_R 0.148 SAFE 0.112 VALUES_R 0.174

TRUST 0.198 SKIP 0.131 GRAFFITI 0.299

DISRESPE 0.093

COVARIANCE COVERAGE OF DATA

It is useful to compare this output to frequency counts from the data file used for initial data management in Stata, SAS or SPSS.

This ensures that the variables were correctly pulled in to Mplus.

Minimum covariance coverage value 0.100

UNIVARIATE PROPORTIONS AND COUNTS FOR CATEGORICAL VARIABLES

CLOSEKNI

Category 1 0.070 90.618

Category 2 0.490 632.358

Category 3 0.067 86.594

Category 4 0.310 400.443

Category 5 0.063 80.987

ADULTS

Category 1 0.090 116.224

Category 2 0.566 730.767

Category 3 0.103 132.685

Category 4 0.220 283.708

Category 5 0.021 27.616

HELP

Category 1 0.108 139.454

Category 2 0.663 855.474

Category 3 0.062 79.917

Category 4 0.143 184.927

Category 5 0.024 31.229

ALONG_R

Category 1 0.086 111.102

Category 2 0.654 843.740

Category 3 0.084 107.866

Category 4 0.166 214.884

Category 5 0.010 13.409

SAFE

Category 1 0.105 135.119

Category 2 0.638 824.239

Category 3 0.090 116.625

Category 4 0.143 184.433

Category 5 0.024 30.585

VALUES_R

Category 1 0.028 36.595

Category 2 0.427 551.799

Category 3 0.115 148.123

Category 4 0.397 512.733

Category 5 0.032 41.750

TRUST

Category 1 0.072 92.562

Category 2 0.597 771.164

Category 3 0.078 101.265

Category 4 0.215 277.510

Category 5 0.038 48.499

SKIP

Category 1 0.200 258.256

Category 2 0.407 525.388

Category 3 0.059 75.551

Category 4 0.243 313.026

Category 5 0.091 117.535

GRAFFITI

Category 1 0.385 496.057

Category 2 0.367 473.343

Category 3 0.037 47.379

Category 4 0.152 196.587

Category 5 0.059 76.389

DISRESPE

Category 1 0.141 181.865

Category 2 0.439 566.717

Category 3 0.092 118.092

Category 4 0.230 296.802

Category 5 0.098 126.280

Note: Thresholds are one component that is estimated when the models include categorical indicators. In this case, the thresholds correspond to the negative cumulative probit for the ordinal response variable when all factors are zero.

SAMPLE STATISTICS

ESTIMATED SAMPLE STATISTICS

MEANS/INTERCEPTS/THRESHOLDS

CLOSEKNI CLOSEKNI CLOSEKNI CLOSEKNI ADULTS$1

________ ________ ________ ________ ________

1 -1.555 0.162 0.346 1.636 -1.541

MEANS/INTERCEPTS/THRESHOLDS

ADULTS$2 ADULTS$3 ADULTS$4 HELP$1 HELP$2

________ ________ ________ ________ ________

1 0.461 0.818 2.341 -1.327 0.805

MEANS/INTERCEPTS/THRESHOLDS

HELP$3 HELP$4 ALONG_R$ ALONG_R$ ALONG_R$

________ ________ ________ ________ ________

1 1.051 2.134 -1.467 0.714 1.025

MEANS/INTERCEPTS/THRESHOLDS

ALONG_R$ SAFE$1 SAFE$2 SAFE$3 SAFE$4

________ ________ ________ ________ ________

1 2.507 -1.334 0.688 1.020 2.095

MEANS/INTERCEPTS/THRESHOLDS

VALUES_R VALUES_R VALUES_R VALUES_R TRUST$1

________ ________ ________ ________ ________

1 -2.080 -0.111 0.210 2.027 -1.609

MEANS/INTERCEPTS/THRESHOLDS

TRUST$2 TRUST$3 TRUST$4 SKIP$1 SKIP$2

________ ________ ________ ________ ________

1 0.491 0.754 2.016 -0.895 0.296

MEANS/INTERCEPTS/THRESHOLDS

SKIP$3 SKIP$4 GRAFFITI GRAFFITI GRAFFITI

________ ________ ________ ________ ________

1 0.466 1.443 -0.342 0.816 0.960

MEANS/INTERCEPTS/THRESHOLDS

GRAFFITI DISRESPE DISRESPE DISRESPE DISRESPE

________ ________ ________ ________ ________

1 1.847 -1.123 0.213 0.470 1.366

WITHIN LEVEL VARIANCE/COVARIANCE

These values represent the sample variances and covariances across individuals, within neighborhoods. High values indicate greater levels of shared variance among the items.

CLOSEKNI ADULTS HELP ALONG_R SAFE

________ ________ ________ ________ ________

CLOSEKNI 1.000

ADULTS 0.439 1.000

HELP 0.523 0.465 1.000

ALONG_R 0.202 0.272 0.319 1.000

SAFE 0.400 0.359 0.476 0.248 1.000

VALUES_R 0.113 0.108 0.181 0.304 0.157

TRUST 0.423 0.463 0.545 0.290 0.542

SKIP 0.298 0.286 0.404 0.192 0.278

GRAFFITI 0.221 0.210 0.270 0.157 0.324

DISRESPE 0.268 0.199 0.287 0.144 0.247

WITHIN LEVEL VARIANCE/COVARIANCE

VALUES_R TRUST SKIP GRAFFITI DISRESPE

________ ________ ________ ________ ________

VALUES_R 1.000

TRUST 0.242 1.000

SKIP 0.063 0.344 1.000

GRAFFITI 0.144 0.224 0.515 1.000

DISRESPE 0.123 0.260 0.521 0.510 1.000

These values represent the standardized variance/covariance matrix at the individual level.

WITHIN LEVEL CORRELATION

CLOSEKNI ADULTS HELP ALONG_R SAFE

________ ________ ________ ________ ________

CLOSEKNI 1.000

ADULTS 0.439 1.000

HELP 0.523 0.465 1.000

ALONG_R 0.202 0.272 0.319 1.000

SAFE 0.400 0.359 0.476 0.248 1.000

VALUES_R 0.113 0.108 0.181 0.304 0.157

TRUST 0.423 0.463 0.545 0.290 0.542

SKIP 0.298 0.286 0.404 0.192 0.278

GRAFFITI 0.221 0.210 0.270 0.157 0.324

DISRESPE 0.268 0.199 0.287 0.144 0.247

WITHIN LEVEL CORRELATION

VALUES_R TRUST SKIP GRAFFITI DISRESPE

________ ________ ________ ________ ________

VALUES_R 1.000

TRUST 0.242 1.000

SKIP 0.063 0.344 1.000

GRAFFITI 0.144 0.224 0.515 1.000

DISRESPE 0.123 0.260 0.521 0.510 1.000

These values represent the sample variances and covariances at the neighborhood level. High values indicate greater levels of shared variance among the items. It is important to note that these values differ than those found in the within level variance/covariance matrix.

BETWEEN LEVEL VARIANCE/COVARIANCE

CLOSEKNI ADULTS HELP ALONG_R SAFE

________ ________ ________ ________ ________

CLOSEKNI 0.126

ADULTS 0.161 0.339

HELP 0.121 0.203 0.166

ALONG_R 0.094 0.205 0.145 0.174

SAFE 0.097 0.173 0.133 0.126 0.126

VALUES_R 0.080 0.142 0.097 0.139 0.105

TRUST 0.126 0.233 0.168 0.183 0.165

SKIP 0.090 0.129 0.123 0.117 0.116

GRAFFITI 0.153 0.297 0.226 0.240 0.209

DISRESPE 0.058 0.036 0.063 0.062 0.039

BETWEEN LEVEL VARIANCE/COVARIANCE

VALUES_R TRUST SKIP GRAFFITI DISRESPE

________ ________ ________ ________ ________

VALUES_R 0.210

TRUST 0.135 0.247

SKIP 0.113 0.144 0.151

GRAFFITI 0.213 0.278 0.177 0.426

DISRESPE 0.039 0.065 0.077 0.089 0.102

These values represent the standardardized variance/covariance matrix at the neighborhood level.

BETWEEN LEVEL CORRELATION

CLOSEKNI ADULTS HELP ALONG_R SAFE

________ ________ ________ ________ ________

CLOSEKNI 1.000

ADULTS 0.779 1.000

HELP 0.837 0.858 1.000

ALONG_R 0.638 0.844 0.858 1.000

SAFE 0.773 0.837 0.920 0.854 1.000

VALUES_R 0.493 0.533 0.520 0.730 0.644

TRUST 0.716 0.807 0.829 0.884 0.934

SKIP 0.650 0.569 0.775 0.725 0.841

GRAFFITI 0.660 0.781 0.849 0.882 0.903

DISRESPE 0.516 0.196 0.482 0.469 0.341

BETWEEN LEVEL CORRELATION

VALUES_R TRUST SKIP GRAFFITI DISRESPE

________ ________ ________ ________ ________

VALUES_R 1.000

TRUST 0.593 1.000

SKIP 0.634 0.745 1.000

GRAFFITI 0.711 0.857 0.698 1.000

DISRESPE 0.267 0.408 0.622 0.426 1.000

Given that MPlus calculates all possible factor combinations, error messages commonly appear here. An example of one such error message is shown below. These error messages signal that some of the models (particularly those with 4 or more factors at either level) could not be estimated. This is likely due to insufficient variance in the items to warrant a 4+ factor structure.

STANDARD ERRORS COULD NOT BE COMPUTED.

PROBLEM OCCURRED IN EXPLORATORY FACTOR ANALYSIS

WITH 4 WITHIN FACTOR(S) AND 1 BETWEEN FACTOR(S).

THIS PROBLEM IS MOST LIKELY CAUSED BY THE RESIDUAL VARIANCE OF SAFE

ON THE WITHIN LEVEL CONVERGING TO ZERO.

CHI-SQUARE TEST COULD NOT BE COMPUTED.

PROBLEM OCCURRED IN EXPLORATORY FACTOR ANALYSIS WITH

4 WITHIN FACTOR(S) AND 1 BETWEEN FACTOR(S).

Note: Some of the Mplus output has been eliminated to shorten the document and improve the ease of use.

.

.

.

**.**

EXPLORATORY FACTOR ANALYSIS WITH 1 WITHIN FACTOR(S) AND 1 BETWEEN FACTOR(S):

These are the results of the EFA with 1 within factor and 1 between factor.

Fit statistics and factor loadings are provided separately for each factor configuration. As shown below, factor loadings are provided at each level.

MODEL FIT INFORMATION

Number of Free Parameters 70

Chi-Square Test of Model Fit

Value 1388.598*

Degrees of Freedom 70

P-Value 0.0000

* The chi-square value for MLM, MLMV, MLR, ULSMV, WLSM and WLSMV cannot be used

for chi-square difference testing in the regular way. MLM, MLR and WLSM

chi-square difference testing is described on the Mplus website. MLMV, WLSMV,

and ULSMV difference testing is done using the DIFFTEST option.

RMSEA (Root Mean Square Error Of Approximation)

RMSEA summarizes the extent to which the model is a good approximation of the observed data. Values below 0.05 indicate close fit. Values above 0.10 indicate poor fit.

Estimate 0.121

90 Percent C.I. 0.115 0.126

Probability RMSEA <= .05 0.000

CFI/TLI

CFI 0.749

TLI 0.677

The CFI and TLI are measures of model fit. They have a range from 0 to 1, with higher values indicating better fit.

Chi-Square Test of Model Fit for the Baseline Model

Value 5338.537

Degrees of Freedom 90

P-Value 0.0000

The SRMR is the only value provided separately at the within- and between-level. The SRMR summarizes the mean absolute value of the correlation residuals for each level. Values below 0.10 are generally acceptable, although values smaller than 0.05 are preferred.

SRMR (Standardized Root Mean Square Residual)

Value for Within 0.084

Value for Between 0.068

MINIMUM ROTATION FUNCTION VALUE 3.36977

See Kline (2001) for more information on interpretation of fit indices.

Mplus presents within-level results first.

WITHIN LEVEL RESULTS

GEOMIN ROTATED LOADINGS (* significant at 5% level)

1

________

CLOSEKNI 0.616*

ADULTS 0.572*

These loadings represent the linear combination of variables that make-up a factor. Loadings for EFA are in standard deviation units.

HELP 0.725*

ALONG_R 0.396*

SAFE 0.632*

VALUES_R 0.274*

TRUST 0.699*

SKIP 0.630*

GRAFFITI 0.583*

DISRESPE 0.528*

GEOMIN FACTOR CORRELATIONS (* significant at 5% level)

1

________

1. 1.000

The residual variances are the variances of the items after accounting for all of the variance in the EFA model. Thus, they are the percentage of variance unexplained.

ESTIMATED RESIDUAL VARIANCES

CLOSEKNI ADULTS HELP ALONG_R SAFE

________ ________ ________ ________ ________

1 0.620 0.672 0.474 0.843 0.601

ESTIMATED RESIDUAL VARIANCES

VALUES_R TRUST SKIP GRAFFITI DISRESPE

________ ________ ________ ________ ________

1 0.925 0.512 0.603 0.660 0.721

S.E. GEOMIN ROTATED LOADINGS

1

________

CLOSEKNI 0.016

ADULTS 0.017

HELP 0.011

ALONG_R 0.021

SAFE 0.016

VALUES_R 0.022

TRUST 0.015

SKIP 0.017

GRAFFITI 0.018

DISRESPE 0.015

S.E. GEOMIN FACTOR CORRELATIONS

1

________

1 0.000

S.E. ESTIMATED RESIDUAL VARIANCES

CLOSEKNI ADULTS HELP ALONG_R SAFE

________ ________ ________ ________ ________

1 0.020 0.020 0.015 0.017 0.021

S.E. ESTIMATED RESIDUAL VARIANCES

VALUES_R TRUST SKIP GRAFFITI DISRESPE

________ ________ ________ ________ ________

1 0.012 0.020 0.022 0.021 0.016

Est./S.E. GEOMIN ROTATED LOADINGS

1

________

CLOSEKNI 38.837

ADULTS 33.555

HELP 68.791

ALONG_R 18.995

SAFE 38.548

VALUES_R 12.591

TRUST 47.865

SKIP 36.681

GRAFFITI 32.806

DISRESPE 35.726

Est./S.E. GEOMIN FACTOR CORRELATIONS

1

________

1 0.000

Est./S.E. ESTIMATED RESIDUAL VARIANCES

CLOSEKNI ADULTS HELP ALONG_R SAFE

________ ________ ________ ________ ________

1 31.699 34.414 30.953 51.059 29.020

Est./S.E. ESTIMATED RESIDUAL VARIANCES

VALUES_R TRUST SKIP GRAFFITI DISRESPE

________ ________ ________ ________ ________

1 77.391 25.091 27.912 31.803 46.241

EXPLORATORY FACTOR ANALYSIS WITH 1 WITHIN FACTOR(S) AND 1 BETWEEN FACTOR(S):

MINIMUM ROTATION FUNCTION VALUE 7.04738

Here is the beginning of the between-level results for the model with 1 factor at each level.

BETWEEN LEVEL RESULTS

GEOMIN ROTATED LOADINGS (* significant at 5% level)

1

________

CLOSEKNI 0.797*

ADULTS 0.833*

HELP 0.935*

ALONG_R 0.931*

SAFE 0.972*

VALUES_R 0.668*

TRUST 0.924*

SKIP 0.823*

GRAFFITI 0.917*

DISRESPE 0.462*

GEOMIN FACTOR CORRELATIONS (* significant at 5% level)

1

________

1 1.000

ESTIMATED RESIDUAL VARIANCES

CLOSEKNI ADULTS HELP ALONG_R SAFE

________ ________ ________ ________ ________

1 0.365 0.305 0.127 0.133 0.056

ESTIMATED RESIDUAL VARIANCES

VALUES_R TRUST SKIP GRAFFITI DISRESPE

________ ________ ________ ________ ________

1 0.554 0.147 0.322 0.160 0.786

S.E. GEOMIN ROTATED LOADINGS

1

________

CLOSEKNI 0.072

ADULTS 0.059

HELP 0.043

ALONG_R 0.041

SAFE 0.036

VALUES_R 0.085

TRUST 0.035

SKIP 0.070

GRAFFITI 0.035

DISRESPE 0.125

S.E. GEOMIN FACTOR CORRELATIONS

1

________

1 0.000

S.E. ESTIMATED RESIDUAL VARIANCES

CLOSEKNI ADULTS HELP ALONG_R SAFE

________ ________ ________ ________ ________

1 0.114 0.098 0.080 0.077 0.070

S.E. ESTIMATED RESIDUAL VARIANCES

VALUES_R TRUST SKIP GRAFFITI DISRESPE

________ ________ ________ ________ ________

1 0.114 0.065 0.115 0.064 0.116

Est./S.E. GEOMIN ROTATED LOADINGS

1

________

CLOSEKNI 11.119

ADULTS 14.142

HELP 21.892

ALONG_R 22.481

SAFE 26.882

VALUES_R 7.836

TRUST 26.128

SKIP 11.775

GRAFFITI 26.116

DISRESPE 3.695

Est./S.E. GEOMIN FACTOR CORRELATIONS

1

________

1 0.000

Est./S.E. ESTIMATED RESIDUAL VARIANCES

CLOSEKNI ADULTS HELP ALONG_R SAFE

________ ________ ________ ________ ________

1 3.197 3.108 1.586 1.723 0.791

Est./S.E. ESTIMATED RESIDUAL VARIANCES

VALUES_R TRUST SKIP GRAFFITI DISRESPE

________ ________ ________ ________ ________

1 4.860 2.246 2.796 2.480 6.795

EXPLORATORY FACTOR ANALYSIS WITH 2 WITHIN FACTOR(S) AND 1 BETWEEN FACTOR(S):

MODEL FIT INFORMATION

This is the beginning of the results for a model with 2 within factors and 1 between factor. This solution is presented as our final EFA model, and these results are presented in the paper in Table 4 of the paper.

Number of Free Parameters 79

Chi-Square Test of Model Fit

Value 337.222*

Degrees of Freedom 61

P-Value 0.0000

* The chi-square value for MLM, MLMV, MLR, ULSMV, WLSM and WLSMV cannot be used

for chi-square difference testing in the regular way. MLM, MLR and WLSM

chi-square difference testing is described on the Mplus website. MLMV, WLSMV,

and ULSMV difference testing is done using the DIFFTEST option.

RMSEA (Root Mean Square Error Of Approximation)

Estimate 0.059

90 Percent C.I. 0.053 0.065

Probability RMSEA <= .05 0.007

CFI/TLI

The fit indices show improvement from the initial model (1 within factor 1 between factor).

CFI 0.947

TLI 0.922

Chi-Square Test of Model Fit for the Baseline Model

Value 5338.537

Degrees of Freedom 90

P-Value 0.0000

SRMR (Standardized Root Mean Square Residual)

Value for Within 0.039

Value for Between 0.068

MINIMUM ROTATION FUNCTION VALUE 0.23948

WITHIN LEVEL RESULTS

GEOMIN ROTATED LOADINGS (* significant at 5% level)

1 2

________ ________

These factor loadings suggest a configuration where the first 7 items load on Factor 1 and the 3 remaining items load on Factor 2. As described in the paper, this solution is consistent with prior research on collective efficacy.

CLOSEKNI 0.618* 0.030

ADULTS 0.642* -0.034

HELP 0.735* 0.038

ALONG_R 0.418* -0.008

SAFE 0.630* 0.035

VALUES_R 0.297* -0.015

TRUST 0.773* -0.046

SKIP 0.121* 0.662*

GRAFFITI 0.001 0.711*

DISRESPE -0.010 0.723*

GEOMIN FACTOR CORRELATIONS (* significant at 5% level)

1 2

________ ________

The correlation between the two within-level factors is 0.521.

1 1.000

2 0.521* 1.000

ESTIMATED RESIDUAL VARIANCES

CLOSEKNI ADULTS HELP ALONG_R SAFE

________ ________ ________ ________ ________

1 0.598 0.610 0.429 0.829 0.578

ESTIMATED RESIDUAL VARIANCES

VALUES_R TRUST SKIP GRAFFITI DISRESPE

________ ________ ________ ________ ________

1 0.916 0.438 0.464 0.494 0.485

S.E. GEOMIN ROTATED LOADINGS

1 2

________ ________

CLOSEKNI 0.027 0.038

ADULTS 0.027 0.037

HELP 0.028 0.044

ALONG_R 0.024 0.018

SAFE 0.025 0.039

VALUES_R 0.031 0.040

TRUST 0.030 0.044

SKIP 0.032 0.027

GRAFFITI 0.011 0.018

DISRESPE 0.026 0.025

S.E. GEOMIN FACTOR CORRELATIONS

1 2

________ ________

1 0.000

2 0.036 0.000

S.E. ESTIMATED RESIDUAL VARIANCES

CLOSEKNI ADULTS HELP ALONG_R SAFE

________ ________ ________ ________ ________

1 0.021 0.023 0.016 0.018 0.020

S.E. ESTIMATED RESIDUAL VARIANCES

VALUES_R TRUST SKIP GRAFFITI DISRESPE

________ ________ ________ ________ ________

1 0.013 0.024 0.025 0.024 0.023

Est./S.E. GEOMIN ROTATED LOADINGS

1 2

________ ________

CLOSEKNI 22.626 0.783

ADULTS 24.106 -0.932

HELP 26.480 0.867

ALONG_R 17.676 -0.463

SAFE 25.602 0.916

VALUES_R 9.492 -0.378

TRUST 25.711 -1.056

SKIP 3.850 24.602

GRAFFITI 0.076 40.248

DISRESPE -0.364 29.407

Est./S.E. GEOMIN FACTOR CORRELATIONS

1 2

________ ________

1 0.000

2 14.299 0.000

Est./S.E. ESTIMATED RESIDUAL VARIANCES

CLOSEKNI ADULTS HELP ALONG_R SAFE

________ ________ ________ ________ ________

1 28.964 26.679 26.112 47.234 29.108

Est./S.E. ESTIMATED RESIDUAL VARIANCES

VALUES_R TRUST SKIP GRAFFITI DISRESPE

________ ________ ________ ________ ________

1 72.645 18.625 18.433 20.985 21.147

FACTOR STRUCTURE

1 2

________ ________

CLOSEKNI 0.634 0.352

ADULTS 0.624 0.300

HELP 0.755 0.421

ALONG_R 0.414 0.209

SAFE 0.649 0.363

VALUES_R 0.290 0.140

TRUST 0.748 0.356

SKIP 0.466 0.725

GRAFFITI 0.371 0.711

DISRESPE 0.367 0.718

EXPLORATORY FACTOR ANALYSIS WITH 2 WITHIN FACTOR(S) AND 1 BETWEEN FACTOR(S):

MINIMUM ROTATION FUNCTION VALUE 7.04741

BETWEEN LEVEL RESULTS

GEOMIN ROTATED LOADINGS (* significant at 5% level)

1

________

CLOSEKNI 0.797*

ADULTS 0.833*

HELP 0.935*

The between-level factor loadings remain the same as the previous 1 factor between model (as expected).

ALONG_R 0.931*

SAFE 0.972*

VALUES_R 0.668*

TRUST 0.924*

SKIP 0.823*

GRAFFITI 0.917*

DISRESPE 0.462*

GEOMIN FACTOR CORRELATIONS (* significant at 5% level)

1

________

1 1.000

ESTIMATED RESIDUAL VARIANCES

CLOSEKNI ADULTS HELP ALONG_R SAFE

________ ________ ________ ________ ________

1 0.365 0.305 0.127 0.133 0.056

ESTIMATED RESIDUAL VARIANCES

VALUES_R TRUST SKIP GRAFFITI DISRESPE

________ ________ ________ ________ ________

1 0.554 0.147 0.322 0.160 0.786

S.E. GEOMIN ROTATED LOADINGS

1

________

CLOSEKNI 0.072

ADULTS 0.059

HELP 0.043

ALONG_R 0.041

SAFE 0.036

VALUES_R 0.085

TRUST 0.035

SKIP 0.070

GRAFFITI 0.035

DISRESPE 0.125

S.E. GEOMIN FACTOR CORRELATIONS

1

________

1 0.000

S.E. ESTIMATED RESIDUAL VARIANCES

CLOSEKNI ADULTS HELP ALONG_R SAFE

________ ________ ________ ________ ________

1 0.114 0.098 0.080 0.077 0.070

S.E. ESTIMATED RESIDUAL VARIANCES

VALUES_R TRUST SKIP GRAFFITI DISRESPE

________ ________ ________ ________ ________

1 0.114 0.065 0.115 0.064 0.116

Est./S.E. GEOMIN ROTATED LOADINGS

1

________

CLOSEKNI 11.119

ADULTS 14.142

HELP 21.892

ALONG_R 22.481

SAFE 26.882

VALUES_R 7.836

TRUST 26.130

SKIP 11.775

GRAFFITI 26.116

DISRESPE 3.695

Est./S.E. GEOMIN FACTOR CORRELATIONS

1

________

1 0.000

Est./S.E. ESTIMATED RESIDUAL VARIANCES

CLOSEKNI ADULTS HELP ALONG_R SAFE

________ ________ ________ ________ ________

1 3.197 3.108 1.586 1.723 0.791

Est./S.E. ESTIMATED RESIDUAL VARIANCES

VALUES_R TRUST SKIP GRAFFITI DISRESPE

________ ________ ________ ________ ________

1 4.860 2.246 2.796 2.480 6.795

Some of the Mplus output has been eliminated to shorten the document and improve the ease of use.

.

.

.

**.**

EXPLORATORY FACTOR ANALYSIS WITH 2 WITHIN FACTOR(S) AND 2 BETWEEN FACTOR(S):

MODEL FIT INFORMATION

This is the beginning of the results for a model with 2 within factors and 2 between factors.

Number of Free Parameters 88

Chi-Square Test of Model Fit

Value 331.008*

Degrees of Freedom 52

P-Value 0.0000

* The chi-square value for MLM, MLMV, MLR, ULSMV, WLSM and WLSMV cannot be used

for chi-square difference testing in the regular way. MLM, MLR and WLSM

chi-square difference testing is described on the Mplus website. MLMV, WLSMV,

and ULSMV difference testing is done using the DIFFTEST option.

RMSEA (Root Mean Square Error Of Approximation)

Estimate 0.064

90 Percent C.I. 0.058 0.071

Probability RMSEA <= .05 0.000

CFI/TLI

Model fit improves from the 2 within and 1 between model to the 2 within and 2 between model, but this is to be expected due to increase in the number of freed parameters.

Thus, it is also important to examine factor loadings for interpretability when comparing models.

CFI 0.947

TLI 0.908

Chi-Square Test of Model Fit for the Baseline Model

Value 5338.537

Degrees of Freedom 90

P-Value 0.0000

SRMR (Standardized Root Mean Square Residual)

Value for Within 0.039

Value for Between 0.045

MINIMUM ROTATION FUNCTION VALUE 0.23948

WITHIN LEVEL RESULTS

GEOMIN ROTATED LOADINGS (* significant at 5% level)

1 2

________ ________

CLOSEKNI 0.618* 0.030

ADULTS 0.642* -0.034

The within-level factor loadings are identical to the within-level estimates from other models with 2 within factors, as expected.

HELP 0.735* 0.038

ALONG_R 0.418* -0.008

SAFE 0.630* 0.035

VALUES_R 0.297* -0.015

TRUST 0.773* -0.046

SKIP 0.121* 0.662*

GRAFFITI 0.001 0.711*

DISRESPE -0.010 0.723*

GEOMIN FACTOR CORRELATIONS (* significant at 5% level)

1 2

________ ________

1 1.000

2 0.521* 1.000

ESTIMATED RESIDUAL VARIANCES

CLOSEKNI ADULTS HELP ALONG_R SAFE

________ ________ ________ ________ ________

1 0.598 0.610 0.429 0.829 0.578

ESTIMATED RESIDUAL VARIANCES

VALUES_R TRUST SKIP GRAFFITI DISRESPE

________ ________ ________ ________ ________

1 0.916 0.438 0.464 0.494 0.485

S.E. GEOMIN ROTATED LOADINGS

1 2

________ ________

CLOSEKNI 0.027 0.038

ADULTS 0.027 0.037

HELP 0.028 0.044

ALONG_R 0.024 0.018

SAFE 0.025 0.039

VALUES_R 0.031 0.040

TRUST 0.030 0.044

SKIP 0.032 0.027

GRAFFITI 0.011 0.018

DISRESPE 0.026 0.025

S.E. GEOMIN FACTOR CORRELATIONS

1 2

________ ________

1 0.000

2 0.036 0.000

S.E. ESTIMATED RESIDUAL VARIANCES

CLOSEKNI ADULTS HELP ALONG_R SAFE

________ ________ ________ ________ ________

1 0.021 0.023 0.016 0.018 0.020

S.E. ESTIMATED RESIDUAL VARIANCES

VALUES_R TRUST SKIP GRAFFITI DISRESPE

________ ________ ________ ________ ________

1 0.013 0.024 0.025 0.024 0.023

Est./S.E. GEOMIN ROTATED LOADINGS

1 2

________ ________

CLOSEKNI 22.626 0.783

ADULTS 24.106 -0.932

HELP 26.480 0.867

ALONG_R 17.676 -0.463

SAFE 25.602 0.916

VALUES_R 9.492 -0.378

TRUST 25.711 -1.056

SKIP 3.850 24.602

GRAFFITI 0.076 40.248

DISRESPE -0.364 29.407

Est./S.E. GEOMIN FACTOR CORRELATIONS

1 2

________ ________

1 0.000

2 14.299 0.000

Est./S.E. ESTIMATED RESIDUAL VARIANCES

CLOSEKNI ADULTS HELP ALONG_R SAFE

________ ________ ________ ________ ________

1 28.964 26.679 26.112 47.234 29.108

Est./S.E. ESTIMATED RESIDUAL VARIANCES

VALUES_R TRUST SKIP GRAFFITI DISRESPE

________ ________ ________ ________ ________

1 72.645 18.625 18.433 20.984 21.147

FACTOR STRUCTURE

1 2

________ ________

CLOSEKNI 0.634 0.352

ADULTS 0.624 0.300

HELP 0.755 0.421

ALONG_R 0.414 0.209

SAFE 0.649 0.363

VALUES_R 0.290 0.140

TRUST 0.748 0.356

SKIP 0.466 0.725

GRAFFITI 0.371 0.711

DISRESPE 0.367 0.718

EXPLORATORY FACTOR ANALYSIS WITH 2 WITHIN FACTOR(S) AND 2 BETWEEN FACTOR(S):

MINIMUM ROTATION FUNCTION VALUE 0.44136

BETWEEN LEVEL RESULTS

GEOMIN ROTATED LOADINGS (* significant at 5% level)

1 2

________ ________

The between-level factor loadings show that the first nine items load on the first factor, while only one item loads on the second factor (though this loading is not significant).

CLOSEKNI 0.762* 0.085

ADULTS 0.889* -0.110

HELP 0.920* 0.033

ALONG_R 0.921* 0.022

SAFE 0.998* -0.058

VALUES_R 0.676* -0.019

TRUST 0.928* -0.009

SKIP 0.771* 0.142

GRAFFITI 0.916* 0.001

DISRESPE 0.000 1.926

GEOMIN FACTOR CORRELATIONS (* significant at 5% level)

1 2

________ ________

1 1.000

2 0.238* 1.000

ESTIMATED RESIDUAL VARIANCES

CLOSEKNI ADULTS HELP ALONG_R SAFE

________ ________ ________ ________ ________

1 0.381 0.245 0.138 0.141 0.028

ESTIMATED RESIDUAL VARIANCES

VALUES_R TRUST SKIP GRAFFITI DISRESPE

________ ________ ________ ________ ________

1 0.549 0.143 0.334 0.160 -2.709

S.E. GEOMIN ROTATED LOADINGS

1 2

________ ________

CLOSEKNI 0.104 0.184

ADULTS 0.090 0.186

HELP 0.051 0.105

ALONG_R 0.047 0.080

SAFE 0.061 0.116

VALUES_R 0.086 0.086

TRUST 0.032 0.052

SKIP 0.147 0.267

GRAFFITI 0.038 0.026

DISRESPE 0.001 2.860

S.E. GEOMIN FACTOR CORRELATIONS

1 2

________ ________

1 0.000

2 0.356 0.000

S.E. ESTIMATED RESIDUAL VARIANCES

CLOSEKNI ADULTS HELP ALONG_R SAFE

________ ________ ________ ________ ________

1 0.118 0.085 0.074 0.072 0.074

S.E. ESTIMATED RESIDUAL VARIANCES

VALUES_R TRUST SKIP GRAFFITI DISRESPE

________ ________ ________ ________ ________

1 0.113 0.059 0.116 0.065 11.013

Est./S.E. GEOMIN ROTATED LOADINGS

1 2

________ ________

CLOSEKNI 7.313 0.461

ADULTS 9.823 -0.593

HELP 17.996 0.314

ALONG_R 19.786 0.271

SAFE 16.365 -0.497

VALUES_R 7.853 -0.218

TRUST 28.857 -0.181

SKIP 5.233 0.531

GRAFFITI 24.340 0.051

DISRESPE 0.168 0.673

Est./S.E. GEOMIN FACTOR CORRELATIONS

1 2

________ ________

1 0.000

2 0.668 0.000

Est./S.E. ESTIMATED RESIDUAL VARIANCES

CLOSEKNI ADULTS HELP ALONG_R SAFE

________ ________ ________ ________ ________

1 3.227 2.879 1.876 1.947 0.375

Est./S.E. ESTIMATED RESIDUAL VARIANCES

VALUES_R TRUST SKIP GRAFFITI DISRESPE

________ ________ ________ ________ ________

1 4.840 2.432 2.881 2.445 -0.246

FACTOR STRUCTURE

1 2

________ ________

CLOSEKNI 0.783 0.266

ADULTS 0.863 0.101

HELP 0.928 0.252

ALONG_R 0.927 0.241

SAFE 0.984 0.179

VALUES_R 0.671 0.142

TRUST 0.926 0.211

SKIP 0.804 0.325

GRAFFITI 0.917 0.219

DISRESPE 0.458 1.926

**Multilevel Confirmatory Factor Analysis (ML-CFA) Syntax**

Mplus VERSION 7

MUTHEN & MUTHEN

INPUT INSTRUCTIONS

Title:

CFA SPECIFYING 2 FACTORS WITHIN AND 1 BETWEEN

Data:

File is ML_EFA_CFA_06_27.dat ;

Variable:

Names are caseid SAMPID_N hhid pid RSA_TYPE wgtrs

wgtadlt wgtpcg closekni adults help along

safe values trust AB6_8 AB6_9 CLOSEKNI0

skip graffiti disrespe AB8_1 AB8_2 AB9

AB11_1 AB11_2 AB11_3 AB12 AB13 AB14

tractx sample1 ALONG_r VALUES_r AGE_YR sex

RB1 RB2_1 RB2_2 RB2_3 RB2_4 RB2_5

RB2_6 AJ5 movsince HA18_1 ;

Missing are . ;

Here we are using the other half of the split sample for the CFA.

USEOBSERVATIONS = sample1 == 0;

USEVARIABLES = closekni adults help along_r

safe values_r trust skip graffiti disrespe;

CATEGORICAL = closekni adults help along_r

Type = twolevel specifies a multilevel model where the within-level and between-level variance/covariance matrices are separately analyzed.

CLUSTER refers to the cluster variable (tractx = Census tract ID).

Based on the results of the EFA, we estimate a 2-factor structure at the within level, corresponding to “social cohesion” and “informal social control.”

Although not shown here, start values may be needed if the model does not run in a reasonable amount of time. Within level starting values can come from a single-level CFA, where the factor analysis is conducted at only one level and the clustering of observations is accounted for through the TYPE=complex command.

For each factor, one loading must be fixed at 1 to allow for model identification. Here, the first factor loading is fixed to 1 as the default in Mplus.

As noted in the EFA, we are using the WLSMV estimator because we are analyzing data from categorical indicators.

safe values_r trust skip graffiti disrespe;

CLUSTER = tractx;

WEIGHT = WGTADLT;

Analysis:

Type = twolevel;

ESTIMATOR=WLSMV;

Model:

%within%

cohesion by closekni;

cohesion by adults;

cohesion by help;

cohesion by along_r;

cohesion by safe;

cohesion by values_r;

cohesion by trust;

control by skip;

control by graffiti;

Factor variances and covariances are freely estimated at the within level. This is apparent by the lack of constraints imposed on these models.

control by disrespe;

cohesion WITH control;

cohesion;

control;

%between%

Based on the results of the EFA, we are estimating a 1-factor structure at the between level.

Although not shown, starting values can be provided to expedite processing. Start values can be obtained by including SVALUES in the output statement. Between level start values can come from the factor loadings obtained from a multi-level EFA with 1 factor loading on the between-level.

col_eff by closekni adults help along_r

safe values_r trust skip graffiti disrespe;

OUTPUT:

sampstat STDYX Residual;

SAVEDATA:

swmatrix is cfa_swmatrix.dat;

The savedata command asks Mplus to create swmatrix file containing the sample statistics at the within and between levels for the CFA sample. This is useful in reducing computing time in subsequent models using the same sample.

**Multilevel Confirmatory Factor Analysis (ML-CFA) Results**

This is the beginning of the output for our CFA model that is presented in Table 5.

SUMMARY OF ANALYSIS

Number of groups 1

Number of observations 1303

Number of dependent variables 10

Number of independent variables 0

Number of continuous latent variables 3

Observed dependent variables

Binary and ordered categorical (ordinal)

CLOSEKNI ADULTS HELP ALONG_R SAFE VALUES_R

TRUST SKIP GRAFFITI DISRESPE

Continuous latent variables

COHESION CONTROL COL_EFF

Variables with special functions

Cluster variable TRACTX

Weight variable (cluster-size scaling)

WGTADLT

Estimator WLSMV

Optimization Specifications for the Quasi-Newton Algorithm for

Continuous Outcomes

Maximum number of iterations 1000

Convergence criterion 0.100D-05

Optimization Specifications for the EM Algorithm

Maximum number of iterations 500

Convergence criteria

Loglikelihood change 0.100D-02

Relative loglikelihood change 0.100D-05

Derivative 0.100D-02

Optimization Specifications for the M step of the EM Algorithm for

Categorical Latent variables

Number of M step iterations 1

M step convergence criterion 0.100D-02

Basis for M step termination ITERATION

Optimization Specifications for the M step of the EM Algorithm for

Censored, Binary or Ordered Categorical (Ordinal), Unordered

Categorical (Nominal) and Count Outcomes

Number of M step iterations 1

M step convergence criterion 0.100D-02

Basis for M step termination ITERATION

Maximum value for logit thresholds 10

Minimum value for logit thresholds -10

Minimum expected cell size for chi-square 0.100D-01

Maximum number of iterations for H1 2000

Convergence criterion for H1 0.100D-03

Optimization algorithm FS

Integration Specifications

Type STANDARD

Number of integration points 7

Dimensions of numerical integration 2

Adaptive quadrature ON

Link PROBIT

Cholesky ON

Input data file(s)

ML_EFA_CFA_06_27.dat

Input data format FREE

These ICCs represent the between-level variance divided by the total variance for each item. Near-zero ICCs suggest minimal neighborhood-based associations.

SUMMARY OF DATA

Number of clusters 65

Average cluster size 20.046

Estimated Intraclass Correlations for the Y Variables

Intraclass Intraclass Intraclass

Variable Correlation Variable Correlation Variable Correlation

CLOSEKNI 0.121 ADULTS 0.216 HELP 0.174

ALONG_R 0.178 SAFE 0.089 VALUES_R 0.114

TRUST 0.254 SKIP 0.125 GRAFFITI 0.273

DISRESPE 0.090

COVARIANCE COVERAGE OF DATA

Minimum covariance coverage value 0.100

UNIVARIATE PROPORTIONS AND COUNTS FOR CATEGORICAL VARIABLES

CLOSEKNI

It is useful to compare this output to frequency counts from the data file used for initial data management in Stata, SAS or SPSS.

This ensures that the variables were correctly pulled in to Mplus.

Category 1 0.063 82.257

Category 2 0.456 594.495

Category 3 0.059 76.525

Category 4 0.378 492.565

Category 5 0.044 57.158

ADULTS

Category 1 0.065 84.773

Category 2 0.542 706.162

Category 3 0.128 167.001

Category 4 0.241 314.254

Category 5 0.024 30.810

HELP

Category 1 0.104 135.301

Category 2 0.660 859.470

Category 3 0.063 82.294

Category 4 0.149 194.529

Category 5 0.024 31.405

ALONG_R

Category 1 0.085 110.708

Category 2 0.596 776.644

Category 3 0.096 124.768

Category 4 0.198 258.423

Category 5 0.025 32.457

SAFE

Category 1 0.085 111.263

Category 2 0.623 812.207

Category 3 0.106 137.679

Category 4 0.152 198.629

Category 5 0.033 43.222

VALUES_R

Category 1 0.036 46.529

Category 2 0.455 593.188

Category 3 0.122 159.053

Category 4 0.347 451.771

Category 5 0.040 52.458

TRUST

Category 1 0.043 56.042

Category 2 0.607 791.541

Category 3 0.118 153.238

Category 4 0.203 265.062

Category 5 0.028 37.118

SKIP

Category 1 0.217 282.169

Category 2 0.390 508.143

Category 3 0.063 81.787

Category 4 0.236 307.307

Category 5 0.095 123.595

GRAFFITI

Category 1 0.388 505.255

Category 2 0.345 449.308

Category 3 0.046 59.931

Category 4 0.160 208.419

Category 5 0.061 80.087

DISRESPE

Category 1 0.168 218.540

Category 2 0.393 512.093

Category 3 0.103 134.355

Note: Thresholds are a component of the estimation of models with categorical indicators. Thresholds refer to the amount of the distribution of a latent, underlying continuous version of each ordered categorical item must respond in a certain category of the observed ordinal item.

Category 4 0.247 321.480

Category 5 0.089 116.531

SAMPLE STATISTICS

ESTIMATED SAMPLE STATISTICS

MEANS/INTERCEPTS/THRESHOLDS

CLOSEKNI CLOSEKNI CLOSEKNI CLOSEKNI ADULTS$1

________ ________ ________ ________ ________

1 -1.631 0.056 0.215 1.820 -1.707

MEANS/INTERCEPTS/THRESHOLDS

ADULTS$2 ADULTS$3 ADULTS$4 HELP$1 HELP$2

________ ________ ________ ________ ________

1 0.311 0.719 2.214 -1.389 0.789

MEANS/INTERCEPTS/THRESHOLDS

HELP$3 HELP$4 ALONG_R$ ALONG_R$ ALONG_R$

________ ________ ________ ________ ________

1 1.032 2.161 -1.514 0.539 0.857

MEANS/INTERCEPTS/THRESHOLDS

ALONG_R$ SAFE$1 SAFE$2 SAFE$3 SAFE$4

________ ________ ________ ________ ________

1 2.132 -1.429 0.585 0.944 1.929

MEANS/INTERCEPTS/THRESHOLDS

VALUES_R VALUES_R VALUES_R VALUES_R TRUST$1

________ ________ ________ ________ ________

1 -1.909 -0.013 0.317 1.859 -1.981

MEANS/INTERCEPTS/THRESHOLDS

TRUST$2 TRUST$3 TRUST$4 SKIP$1 SKIP$2

________ ________ ________ ________ ________

1 0.462 0.864 2.172 -0.831 0.299

MEANS/INTERCEPTS/THRESHOLDS

SKIP$3 SKIP$4 GRAFFITI GRAFFITI GRAFFITI

________ ________ ________ ________ ________

1 0.480 1.413 -0.302 0.753 0.919

MEANS/INTERCEPTS/THRESHOLDS

GRAFFITI DISRESPE DISRESPE DISRESPE DISRESPE

________ ________ ________ ________ ________

1 1.783 -1.003 0.162 0.445 1.415

These values represent the sample variances and covariances across individuals, within neighborhoods. High values indicate greater levels of shared variance among the items.

WITHIN LEVEL VARIANCE/COVARIANCE

CLOSEKNI ADULTS HELP ALONG_R SAFE

________ ________ ________ ________ ________

CLOSEKNI 1.000

ADULTS 0.455 1.000

HELP 0.453 0.475 1.000

ALONG_R 0.201 0.314 0.391 1.000

SAFE 0.407 0.412 0.444 0.243 1.000

VALUES_R 0.194 0.058 0.137 0.335 0.125

TRUST 0.389 0.397 0.516 0.329 0.414

SKIP 0.252 0.157 0.235 0.169 0.401

GRAFFITI 0.202 0.273 0.289 0.246 0.377

DISRESPE 0.290 0.188 0.273 0.224 0.276

WITHIN LEVEL VARIANCE/COVARIANCE

VALUES_R TRUST SKIP GRAFFITI DISRESPE

________ ________ ________ ________ ________

VALUES_R 1.000

TRUST 0.207 1.000

SKIP 0.166 0.373 1.000

GRAFFITI 0.152 0.348 0.581 1.000

DISRESPE 0.113 0.296 0.420 0.459 1.000

These values represent the standardardized variance/covariance matrix at the individual level.

WITHIN LEVEL CORRELATION

CLOSEKNI ADULTS HELP ALONG_R SAFE

________ ________ ________ ________ ________

CLOSEKNI 1.000

ADULTS 0.455 1.000

HELP 0.453 0.475 1.000

ALONG_R 0.201 0.314 0.391 1.000

SAFE 0.407 0.412 0.444 0.243 1.000

VALUES_R 0.194 0.058 0.137 0.335 0.125

TRUST 0.389 0.397 0.516 0.329 0.414

SKIP 0.252 0.157 0.235 0.169 0.401

GRAFFITI 0.202 0.273 0.289 0.246 0.377

DISRESPE 0.290 0.188 0.273 0.224 0.276

WITHIN LEVEL CORRELATION

VALUES_R TRUST SKIP GRAFFITI DISRESPE

________ ________ ________ ________ ________

VALUES_R 1.000

TRUST 0.207 1.000

SKIP 0.166 0.373 1.000

GRAFFITI 0.152 0.348 0.581 1.000

DISRESPE 0.113 0.296 0.420 0.459 1.000

These values represent the sample variances and covariances across neighborhoods. High values indicate greater levels of shared variance among the items.

BETWEEN LEVEL VARIANCE/COVARIANCE

CLOSEKNI ADULTS HELP ALONG_R SAFE

________ ________ ________ ________ ________

CLOSEKNI 0.137

ADULTS 0.134 0.275

HELP 0.109 0.183 0.211

ALONG_R 0.093 0.160 0.168 0.216

SAFE 0.068 0.102 0.110 0.097 0.097

VALUES_R 0.071 0.115 0.121 0.132 0.074

TRUST 0.151 0.224 0.220 0.209 0.153

SKIP 0.094 0.100 0.068 0.089 0.072

GRAFFITI 0.166 0.264 0.206 0.231 0.142

DISRESPE 0.058 0.055 0.055 0.035 0.018

BETWEEN LEVEL VARIANCE/COVARIANCE

VALUES_R TRUST SKIP GRAFFITI DISRESPE

________ ________ ________ ________ ________

VALUES_R 0.128

TRUST 0.150 0.341

SKIP 0.079 0.125 0.143

GRAFFITI 0.149 0.292 0.176 0.376

DISRESPE 0.042 0.032 0.035 0.046 0.099

These values represent the standardardized variance/covariance matrix at the neighborhood level.

BETWEEN LEVEL CORRELATION

CLOSEKNI ADULTS HELP ALONG_R SAFE

________ ________ ________ ________ ________

CLOSEKNI 1.000

ADULTS 0.691 1.000

HELP 0.642 0.760 1.000

ALONG_R 0.540 0.657 0.789 1.000

SAFE 0.592 0.625 0.767 0.667 1.000

VALUES_R 0.537 0.616 0.738 0.796 0.662

TRUST 0.698 0.732 0.820 0.769 0.842

SKIP 0.670 0.504 0.392 0.507 0.611

GRAFFITI 0.731 0.820 0.733 0.809 0.741

DISRESPE 0.498 0.332 0.384 0.241 0.182

BETWEEN LEVEL CORRELATION

VALUES_R TRUST SKIP GRAFFITI DISRESPE

________ ________ ________ ________ ________

VALUES_R 1.000

TRUST 0.716 1.000

SKIP 0.587 0.565 1.000

GRAFFITI 0.678 0.814 0.759 1.000

DISRESPE 0.374 0.175 0.292 0.239 1.000

THE MODEL ESTIMATION TERMINATED NORMALLY

MODEL FIT INFORMATION

Number of Free Parameters 71

Chi-Square Test of Model Fit

Value 629.816*

Degrees of Freedom 69

P-Value 0.0000

* The chi-square value for MLM, MLMV, MLR, ULSMV, WLSM and WLSMV cannot be used

for chi-square difference testing in the regular way. MLM, MLR and WLSM

chi-square difference testing is described on the Mplus website. MLMV, WLSMV,

and ULSMV difference testing is done using the DIFFTEST option.

RMSEA (Root Mean Square Error Of Approximation)

RMSEA summarizes the extent to which the model is a good approximation of the observed data. Values below 0.05 indicate close fit. Values above 0.10 indicate poor fit.

The CFI and TLI are measures of model fit. They have a range from 0 to 1, with higher values indicating better fit.

Estimate 0.079

CFI/TLI

CFI 0.903

TLI 0.874

Chi-Square Test of Model Fit for the Baseline Model

Value 5899.990

Degrees of Freedom 90

P-Value 0.0000

SRMR (Standardized Root Mean Square Residual)

See Kline (2001) for more information on interpretation of fit indices.

The SRMR is the only value provided separately at the within- and between-level. The SRMR summarizes the mean absolute value of the correlation residuals for each level. Values below 0.10 are generally acceptable, although values smaller than 0.05 are preferred.

Value for Within 0.054

Value for Between 0.073

WRMR (Weighted Root Mean Square Residual)

Value 1.694

MODEL RESULTS

These are the unstandardized model results. Each estimate represents a factor loading or “lambda” coefficient. Each loading can be interpreted similarly to a beta coefficient from a regression analysis.

By default, Mplus constrains the first factor loading for each factor to 1.

Two-Tailed

Estimate S.E. Est./S.E. P-Value

Within Level

COHESION BY

CLOSEKNI 1.000 0.000 999.000 999.000

ADULTS 1.023 0.081 12.692 0.000

HELP 1.236 0.065 19.098 0.000

ALONG_R 0.677 0.043 15.724 0.000

SAFE 1.074 0.054 19.834 0.000

VALUES_R 0.348 0.030 11.413 0.000

TRUST 1.169 0.069 16.889 0.000

CONTROL BY

SKIP 1.000 0.000 999.000 999.000

GRAFFITI 1.146 0.082 13.908 0.000

DISRESPE 0.738 0.055 13.434 0.000

COHESION WITH

CONTROL 0.524 0.037 14.194 0.000

Variances

COHESION 0.633 0.055 11.558 0.000

CONTROL 1.104 0.096 11.450 0.000

Between Level

COL_EFF BY

CLOSEKNI 1.000 0.000 999.000 999.000

ADULTS 1.520 0.399 3.810 0.000

HELP 1.505 0.392 3.837 0.000

ALONG_R 1.193 0.387 3.081 0.002

SAFE 0.916 0.235 3.905 0.000

VALUES_R 0.817 0.248 3.291 0.001

TRUST 1.951 0.517 3.777 0.000

SKIP 0.999 0.249 4.012 0.000

GRAFFITI 2.433 0.566 4.302 0.000

DISRESPE 0.384 0.155 2.469 0.014

Thresholds

CLOSEKNI$1 -2.084 0.089 -23.394 0.000

CLOSEKNI$2 0.071 0.072 0.983 0.325

CLOSEKNI$3 0.274 0.076 3.624 0.000

CLOSEKNI$4 2.326 0.096 24.351 0.000

ADULTS$1 -2.202 0.127 -17.316 0.000

ADULTS$2 0.401 0.103 3.905 0.000

ADULTS$3 0.928 0.098 9.505 0.000

ADULTS$4 2.855 0.147 19.453 0.000

HELP$1 -1.948 0.114 -17.152 0.000

HELP$2 1.106 0.099 11.202 0.000

HELP$3 1.447 0.099 14.648 0.000

HELP$4 3.030 0.106 28.615 0.000

ALONG_R$1 -1.719 0.094 -18.200 0.000

ALONG_R$2 0.612 0.084 7.317 0.000

ALONG_R$3 0.973 0.088 11.082 0.000

ALONG_R$4 2.421 0.111 21.729 0.000

SAFE$1 -1.880 0.079 -23.660 0.000

SAFE$2 0.769 0.074 10.457 0.000

SAFE$3 1.242 0.077 16.102 0.000

SAFE$4 2.537 0.089 28.489 0.000

VALUES_R$1 -1.981 0.073 -26.998 0.000

VALUES_R$2 -0.013 0.058 -0.226 0.821

VALUES_R$3 0.329 0.059 5.624 0.000

VALUES_R$4 1.929 0.074 26.111 0.000

TRUST$1 -2.706 0.158 -17.082 0.000

TRUST$2 0.631 0.119 5.321 0.000

TRUST$3 1.180 0.122 9.708 0.000

TRUST$4 2.967 0.137 21.616 0.000

SKIP$1 -1.205 0.090 -13.379 0.000

SKIP$2 0.434 0.091 4.785 0.000

SKIP$3 0.696 0.089 7.835 0.000

SKIP$4 2.049 0.110 18.580 0.000

GRAFFITI$1 -0.473 0.147 -3.223 0.001

GRAFFITI$2 1.180 0.154 7.658 0.000

GRAFFITI$3 1.439 0.152 9.473 0.000

GRAFFITI$4 2.791 0.160 17.463 0.000

DISRESPE$1 -1.270 0.073 -17.353 0.000

DISRESPE$2 0.205 0.066 3.129 0.002

DISRESPE$3 0.563 0.066 8.552 0.000

DISRESPE$4 1.791 0.073 24.481 0.000

Variances

COL_EFF 0.134 0.064 2.104 0.035

Residual Variances

CLOSEKNI 0.090 0.030 3.044 0.002

ADULTS 0.146 0.041 3.607 0.000

HELP 0.110 0.037 2.952 0.003

ALONG_R 0.088 0.027 3.195 0.001

SAFE 0.055 0.022 2.489 0.013

VALUES_R 0.048 0.019 2.538 0.011

TRUST 0.125 0.045 2.751 0.006

SKIP 0.167 0.046 3.669 0.000

GRAFFITI 0.127 0.069 1.849 0.064

DISRESPE 0.139 0.042 3.329 0.001

These are the standardized model results, which are presented in Table 5. Each loading can be interpreted similarly to a regression coefficient in standard deviation units.

STANDARDIZED MODEL RESULTS

STDYX Standardization

Two-Tailed

Estimate S.E. Est./S.E. P-Value

Within Level

COHESION BY

CLOSEKNI 0.622 0.016 37.736 0.000

ADULTS 0.631 0.019 32.775 0.000

HELP 0.701 0.014 49.943 0.000

ALONG_R 0.474 0.017 28.612 0.000

SAFE 0.649 0.015 42.788 0.000

VALUES_R 0.266 0.018 14.625 0.000

TRUST 0.681 0.015 46.453 0.000

CONTROL BY

SKIP 0.724 0.015 48.186 0.000

GRAFFITI 0.769 0.017 45.883 0.000

DISRESPE 0.613 0.020 31.102 0.000

COHESION WITH

CONTROL 0.627 0.020 32.147 0.000

Variances

COHESION 1.000 0.000 999.000 999.000

CONTROL 1.000 0.000 999.000 999.000

Between Level

COL_EFF BY

CLOSEKNI 0.774 0.073 10.632 0.000

ADULTS 0.824 0.053 15.645 0.000

HELP 0.857 0.054 15.775 0.000

ALONG_R 0.828 0.066 12.585 0.000

SAFE 0.819 0.063 12.946 0.000

VALUES_R 0.807 0.070 11.501 0.000

TRUST 0.897 0.053 17.049 0.000

SKIP 0.667 0.082 8.152 0.000

GRAFFITI 0.928 0.038 24.268 0.000

DISRESPE 0.353 0.127 2.782 0.005

Thresholds

CLOSEKNI$1 -1.631 0.069 -23.640 0.000

CLOSEKNI$2 0.056 0.057 0.985 0.325

CLOSEKNI$3 0.215 0.059 3.645 0.000

CLOSEKNI$4 1.820 0.072 25.303 0.000

ADULTS$1 -1.707 0.093 -18.413 0.000

ADULTS$2 0.311 0.081 3.847 0.000

ADULTS$3 0.719 0.078 9.239 0.000

ADULTS$4 2.214 0.119 18.559 0.000

HELP$1 -1.389 0.079 -17.681 0.000

HELP$2 0.789 0.071 11.060 0.000

HELP$3 1.032 0.071 14.481 0.000

HELP$4 2.161 0.077 27.891 0.000

ALONG_R$1 -1.514 0.083 -18.300 0.000

ALONG_R$2 0.539 0.074 7.296 0.000

ALONG_R$3 0.857 0.077 11.056 0.000

ALONG_R$4 2.132 0.100 21.254 0.000

SAFE$1 -1.429 0.062 -23.224 0.000

SAFE$2 0.585 0.055 10.708 0.000

SAFE$3 0.944 0.057 16.599 0.000

SAFE$4 1.929 0.062 30.871 0.000

VALUES_R$1 -1.909 0.071 -26.889 0.000

VALUES_R$2 -0.013 0.056 -0.226 0.821

VALUES_R$3 0.317 0.056 5.640 0.000

VALUES_R$4 1.859 0.070 26.486 0.000

TRUST$1 -1.981 0.114 -17.372 0.000

TRUST$2 0.462 0.087 5.335 0.000

TRUST$3 0.864 0.088 9.808 0.000

TRUST$4 2.172 0.104 20.796 0.000

SKIP$1 -0.831 0.065 -12.846 0.000

SKIP$2 0.299 0.061 4.908 0.000

SKIP$3 0.480 0.059 8.196 0.000

SKIP$4 1.413 0.066 21.467 0.000

GRAFFITI$1 -0.302 0.092 -3.285 0.001

GRAFFITI$2 0.753 0.101 7.466 0.000

GRAFFITI$3 0.919 0.100 9.224 0.000

GRAFFITI$4 1.783 0.108 16.470 0.000

DISRESPE$1 -1.003 0.055 -18.204 0.000

DISRESPE$2 0.162 0.052 3.128 0.002

DISRESPE$3 0.445 0.052 8.593 0.000

DISRESPE$4 1.415 0.054 26.211 0.000

Variances

COL_EFF 1.000 0.000 999.000 999.000

Residual Variances

CLOSEKNI 0.401 0.113 3.555 0.000

ADULTS 0.320 0.087 3.689 0.000

HELP 0.265 0.093 2.850 0.004

ALONG_R 0.314 0.109 2.882 0.004

SAFE 0.329 0.104 3.173 0.002

VALUES_R 0.349 0.113 3.088 0.002

TRUST 0.196 0.094 2.080 0.038

SKIP 0.555 0.109 5.082 0.000

GRAFFITI 0.138 0.071 1.943 0.052

DISRESPE 0.875 0.090 9.781 0.000

R-SQUARE

Within Level

Observed Two-Tailed Scale

Variable Estimate S.E. Est./S.E. P-Value Factors

CLOSEKNI 0.387 0.021 18.868 0.000 0.783

ADULTS 0.398 0.024 16.388 0.000 0.776

HELP 0.491 0.020 24.971 0.000 0.713

ALONG_R 0.225 0.016 14.306 0.000 0.880

SAFE 0.422 0.020 21.394 0.000 0.760

VALUES_R 0.071 0.010 7.313 0.000 0.964

TRUST 0.464 0.020 23.227 0.000 0.732

SKIP 0.525 0.022 24.093 0.000 0.689

GRAFFITI 0.592 0.026 22.941 0.000 0.639

DISRESPE 0.376 0.024 15.551 0.000 0.790

Between Level

Observed Two-Tailed

Variable Estimate S.E. Est./S.E. P-Value

CLOSEKNI 0.599 0.113 5.316 0.000

ADULTS 0.680 0.087 7.822 0.000

HELP 0.735 0.093 7.887 0.000

ALONG_R 0.686 0.109 6.292 0.000

SAFE 0.671 0.104 6.473 0.000

VALUES_R 0.651 0.113 5.750 0.000

TRUST 0.804 0.094 8.525 0.000

SKIP 0.445 0.109 4.076 0.000

GRAFFITI 0.862 0.071 12.134 0.000

DISRESPE 0.125 0.090 1.391 0.164

QUALITY OF NUMERICAL RESULTS

Condition Number for the Information Matrix 0.130E-03

(ratio of smallest to largest eigenvalue)

RESIDUAL OUTPUT

ESTIMATED MODEL AND RESIDUALS (OBSERVED - ESTIMATED)

Model Estimated Means/Intercepts/Thresholds

CLOSEKNI CLOSEKNI CLOSEKNI CLOSEKNI ADULTS$1

________ ________ ________ ________ ________

1 -1.631 0.056 0.215 1.820 -1.707

Model Estimated Means/Intercepts/Thresholds

ADULTS$2 ADULTS$3 ADULTS$4 HELP$1 HELP$2

________ ________ ________ ________ ________

1 0.311 0.719 2.214 -1.389 0.789

Model Estimated Means/Intercepts/Thresholds

HELP$3 HELP$4 ALONG_R$ ALONG_R$ ALONG_R$

________ ________ ________ ________ ________

1 1.032 2.161 -1.514 0.539 0.857

Model Estimated Means/Intercepts/Thresholds

ALONG_R$ SAFE$1 SAFE$2 SAFE$3 SAFE$4

________ ________ ________ ________ ________

1 2.132 -1.429 0.585 0.944 1.929

Model Estimated Means/Intercepts/Thresholds

VALUES_R VALUES_R VALUES_R VALUES_R TRUST$1

________ ________ ________ ________ ________

1 -1.909 -0.013 0.317 1.859 -1.981

Model Estimated Means/Intercepts/Thresholds

TRUST$2 TRUST$3 TRUST$4 SKIP$1 SKIP$2

________ ________ ________ ________ ________

1 0.462 0.864 2.172 -0.831 0.299

Model Estimated Means/Intercepts/Thresholds

SKIP$3 SKIP$4 GRAFFITI GRAFFITI GRAFFITI

________ ________ ________ ________ ________

1 0.480 1.413 -0.302 0.753 0.919

Model Estimated Means/Intercepts/Thresholds

GRAFFITI DISRESPE DISRESPE DISRESPE DISRESPE

________ ________ ________ ________ ________

1 1.783 -1.003 0.162 0.445 1.415

Residuals for Means/Intercepts/Thresholds

CLOSEKNI CLOSEKNI CLOSEKNI CLOSEKNI ADULTS$1

________ ________ ________ ________ ________

1 0.000 0.000 0.000 0.000 0.000

Residuals for Means/Intercepts/Thresholds

ADULTS$2 ADULTS$3 ADULTS$4 HELP$1 HELP$2

________ ________ ________ ________ ________

1 0.000 0.000 0.000 0.000 0.000

Residuals for Means/Intercepts/Thresholds

HELP$3 HELP$4 ALONG_R$ ALONG_R$ ALONG_R$

________ ________ ________ ________ ________

1 0.000 0.000 0.000 0.000 0.000

Residuals for Means/Intercepts/Thresholds

ALONG_R$ SAFE$1 SAFE$2 SAFE$3 SAFE$4

________ ________ ________ ________ ________

1 0.000 0.000 0.000 0.000 0.000

Residuals for Means/Intercepts/Thresholds

VALUES_R VALUES_R VALUES_R VALUES_R TRUST$1

________ ________ ________ ________ ________

1 0.000 0.000 0.000 0.000 0.000

Residuals for Means/Intercepts/Thresholds

TRUST$2 TRUST$3 TRUST$4 SKIP$1 SKIP$2

________ ________ ________ ________ ________

1 0.000 0.000 0.000 0.000 0.000

Residuals for Means/Intercepts/Thresholds

SKIP$3 SKIP$4 GRAFFITI GRAFFITI GRAFFITI

________ ________ ________ ________ ________

1 0.000 0.000 0.000 0.000 0.000

Residuals for Means/Intercepts/Thresholds

GRAFFITI DISRESPE DISRESPE DISRESPE DISRESPE

________ ________ ________ ________ ________

1 0.000 0.000 0.000 0.000 0.000

Model Estimated Within Level Covariances

CLOSEKNI ADULTS HELP ALONG_R SAFE

________ ________ ________ ________ ________

CLOSEKNI 1.000

ADULTS 0.393 1.000

HELP 0.436 0.442 1.000

ALONG_R 0.295 0.299 0.332 1.000

SAFE 0.404 0.410 0.455 0.308 1.000

VALUES_R 0.166 0.168 0.187 0.126 0.173

TRUST 0.424 0.430 0.477 0.323 0.442

SKIP 0.283 0.287 0.319 0.216 0.295

GRAFFITI 0.301 0.305 0.338 0.229 0.314

DISRESPE 0.239 0.243 0.270 0.182 0.250

Model Estimated Within Level Covariances

VALUES_R TRUST SKIP GRAFFITI DISRESPE

________ ________ ________ ________ ________

VALUES_R 1.000

TRUST 0.181 1.000

SKIP 0.121 0.310 1.000

GRAFFITI 0.129 0.329 0.557 1.000

DISRESPE 0.102 0.262 0.444 0.472 1.000

Residuals for Within Level Covariances

CLOSEKNI ADULTS HELP ALONG_R SAFE

________ ________ ________ ________ ________

CLOSEKNI 0.000

ADULTS 0.062 0.000

HELP 0.016 0.032 0.000

ALONG_R -0.094 0.015 0.058 0.000

SAFE 0.003 0.002 -0.011 -0.065 0.000

VALUES_R 0.028 -0.110 -0.050 0.209 -0.048

TRUST -0.035 -0.033 0.039 0.006 -0.029

SKIP -0.031 -0.130 -0.084 -0.046 0.106

GRAFFITI -0.098 -0.032 -0.050 0.017 0.064

DISRESPE 0.051 -0.055 0.003 0.041 0.026

Residuals for Within Level Covariances

VALUES_R TRUST SKIP GRAFFITI DISRESPE

________ ________ ________ ________ ________

VALUES_R 0.000

TRUST 0.025 0.000

SKIP 0.045 0.063 0.000

GRAFFITI 0.024 0.020 0.024 0.000

DISRESPE 0.011 0.034 -0.024 -0.013 0.000

Model Estimated Within Level Correlations

CLOSEKNI ADULTS HELP ALONG_R SAFE

________ ________ ________ ________ ________

CLOSEKNI 1.000

ADULTS 0.393 1.000

HELP 0.436 0.442 1.000

ALONG_R 0.295 0.299 0.332 1.000

SAFE 0.404 0.410 0.455 0.308 1.000

VALUES_R 0.166 0.168 0.187 0.126 0.173

TRUST 0.424 0.430 0.477 0.323 0.442

SKIP 0.283 0.287 0.319 0.216 0.295

GRAFFITI 0.301 0.305 0.338 0.229 0.314

DISRESPE 0.239 0.243 0.270 0.182 0.250

Model Estimated Within Level Correlations

VALUES_R TRUST SKIP GRAFFITI DISRESPE

________ ________ ________ ________ ________

VALUES_R 1.000

TRUST 0.181 1.000

SKIP 0.121 0.310 1.000

GRAFFITI 0.129 0.329 0.557 1.000

DISRESPE 0.102 0.262 0.444 0.472 1.000

It is important to inspect the correlation residuals for signs of misfit. Correlations with an absolute value of 0.1 or greater should be flagged, and model modifications should be considered (assuming these modifications are consistent with theory).

Residuals for Within Level Correlations

CLOSEKNI ADULTS HELP ALONG_R SAFE

________ ________ ________ ________ ________

CLOSEKNI 0.000

ADULTS 0.062 0.000

HELP 0.016 0.032 0.000

ALONG_R -0.094 0.015 0.058 0.000

SAFE 0.003 0.002 -0.011 -0.065 0.000

VALUES_R 0.028 -0.110 -0.050 0.209 -0.048

TRUST -0.035 -0.033 0.039 0.006 -0.029

SKIP -0.031 -0.130 -0.084 -0.046 0.106

GRAFFITI -0.098 -0.032 -0.050 0.017 0.064

DISRESPE 0.051 -0.055 0.003 0.041 0.026

Residuals for Within Level Correlations

VALUES_R TRUST SKIP GRAFFITI DISRESPE

________ ________ ________ ________ ________

VALUES_R 0.000

TRUST 0.025 0.000

SKIP 0.045 0.063 0.000

GRAFFITI 0.024 0.020 0.024 0.000

DISRESPE 0.011 0.034 -0.024 -0.013 0.000

Model Estimated Between Level Covariances

CLOSEKNI ADULTS HELP ALONG_R SAFE

________ ________ ________ ________ ________

CLOSEKNI 0.137

ADULTS 0.124 0.275

HELP 0.113 0.170 0.211

ALONG_R 0.110 0.166 0.152 0.216

SAFE 0.073 0.110 0.100 0.098 0.097

VALUES_R 0.083 0.125 0.114 0.111 0.074

TRUST 0.150 0.226 0.206 0.202 0.134

SKIP 0.072 0.109 0.099 0.097 0.064

GRAFFITI 0.163 0.246 0.224 0.219 0.145

DISRESPE 0.032 0.048 0.044 0.043 0.028

Model Estimated Between Level Covariances

VALUES_R TRUST SKIP GRAFFITI DISRESPE

________ ________ ________ ________ ________

VALUES_R 0.128

TRUST 0.151 0.341

SKIP 0.073 0.132 0.143

GRAFFITI 0.164 0.298 0.144 0.376

DISRESPE 0.032 0.058 0.028 0.063 0.099

Residuals for Between Level Covariances

CLOSEKNI ADULTS HELP ALONG_R SAFE

________ ________ ________ ________ ________

CLOSEKNI 0.000

ADULTS 0.010 0.000

HELP -0.004 0.013 0.000

ALONG_R -0.017 -0.006 0.017 0.000

SAFE -0.005 -0.008 0.009 -0.002 0.000

VALUES_R -0.012 -0.009 0.008 0.021 0.000

TRUST 0.001 -0.002 0.014 0.007 0.020

SKIP 0.022 -0.009 -0.031 -0.008 0.008

GRAFFITI 0.003 0.018 -0.018 0.011 -0.004

DISRESPE 0.026 0.007 0.012 -0.007 -0.010

Residuals for Between Level Covariances

VALUES_R TRUST SKIP GRAFFITI DISRESPE

________ ________ ________ ________ ________

VALUES_R 0.000

TRUST -0.001 0.000

SKIP 0.007 -0.007 0.000

GRAFFITI -0.016 -0.007 0.033 0.000

DISRESPE 0.010 -0.026 0.007 -0.017 0.000

Model Estimated Between Level Correlations

CLOSEKNI ADULTS HELP ALONG_R SAFE

________ ________ ________ ________ ________

CLOSEKNI 1.000

ADULTS 0.638 1.000

HELP 0.663 0.707 1.000

ALONG_R 0.641 0.683 0.710 1.000

SAFE 0.634 0.675 0.702 0.678 1.000

VALUES_R 0.624 0.665 0.691 0.668 0.661

TRUST 0.694 0.739 0.768 0.743 0.734

SKIP 0.516 0.550 0.572 0.552 0.546

GRAFFITI 0.719 0.765 0.796 0.769 0.761

DISRESPE 0.273 0.291 0.302 0.292 0.289

Model Estimated Between Level Correlations

VALUES_R TRUST SKIP GRAFFITI DISRESPE

________ ________ ________ ________ ________

VALUES_R 1.000

TRUST 0.723 1.000

SKIP 0.538 0.598 1.000

GRAFFITI 0.749 0.832 0.619 1.000

DISRESPE 0.285 0.316 0.235 0.328 1.000

It is important to inspect the correlation residuals for signs of misfit. Correlations with an absolute value of 0.1 or greater should be flagged, and model modifications should be considered (assuming these modifications are consistent with theory).

Residuals for Between Level Correlations

CLOSEKNI ADULTS HELP ALONG_R SAFE

________ ________ ________ ________ ________

CLOSEKNI 0.000

ADULTS 0.053 0.000

HELP -0.021 0.054 0.000

ALONG_R -0.101 -0.025 0.079 0.000

SAFE -0.042 -0.050 0.065 -0.011 0.000

VALUES_R -0.088 -0.049 0.046 0.128 0.001

TRUST 0.004 -0.007 0.052 0.027 0.107

SKIP 0.154 -0.046 -0.180 -0.045 0.065

GRAFFITI 0.012 0.055 -0.063 0.040 -0.020

DISRESPE 0.225 0.041 0.081 -0.051 -0.107

Residuals for Between Level Correlations

VALUES_R TRUST SKIP GRAFFITI DISRESPE

________ ________ ________ ________ ________

VALUES_R 0.000

TRUST -0.007 0.000

SKIP 0.048 -0.033 0.000

GRAFFITI -0.071 -0.019 0.140 0.000

DISRESPE 0.090 -0.141 0.057 -0.089 0.000

SAVEDATA INFORMATION

Within and between sample statistics with Weight matrix

Save file

cfa_swmatrix.dat

Save format Free

DIAGRAM INFORMATION

Mplus diagrams are currently not available for multilevel analysis.

No diagram output was produced.

Beginning Time: 17:02:16

Ending Time: 17:03:40

Elapsed Time: 00:01:24

MUTHEN & MUTHEN

3463 Stoner Ave.

Los Angeles, CA 90066

Tel: (310) 391-9971

Fax: (310) 391-8971

Web: www.StatModel.com

Support: Support@StatModel.com

Copyright (c) 1998-2012 Muthen & Muthen
